# Supplementary figures and images for: High LYRM4-AS1 predicts poor prognosis in patients with glioma and correlates with immune infiltration
Source: PeerJ. 2023 Oct 3;11:e16104. doi: 10.7717/peerj.16104 (PMC10557942; doi:10.7717/peerj.16104)

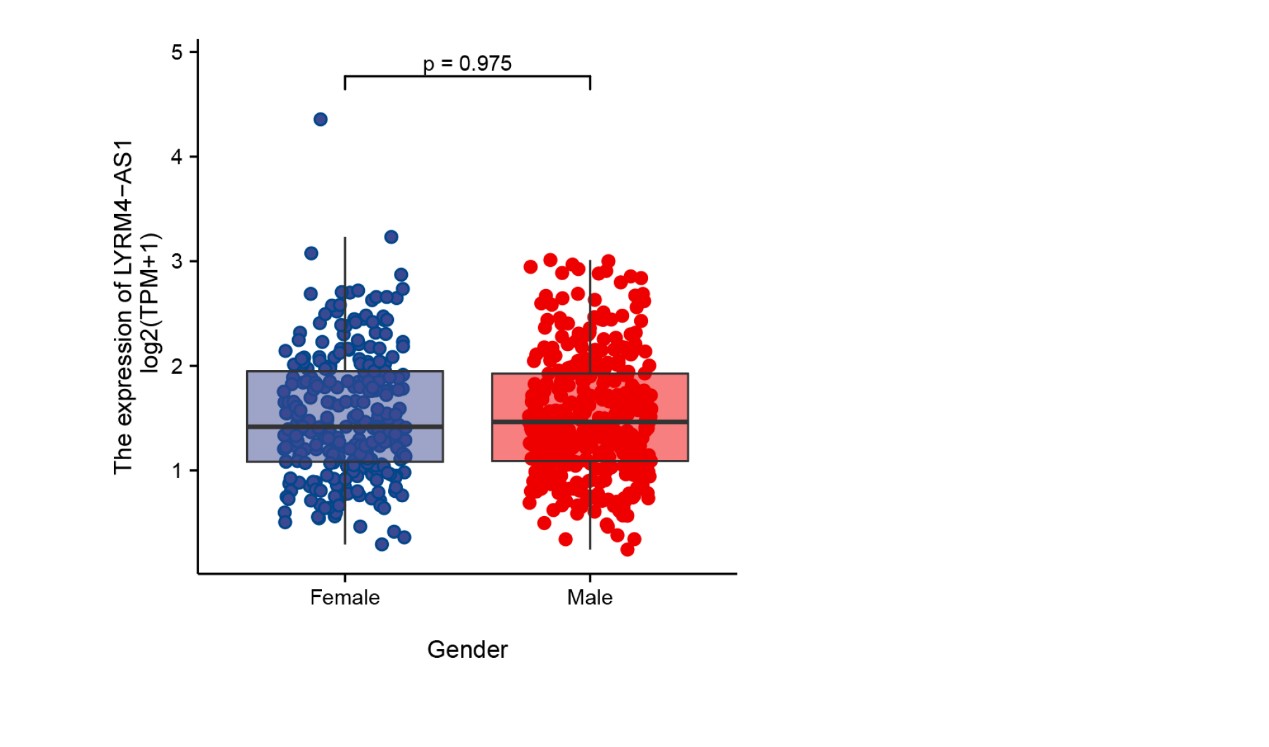

Supplement: Supplemental Information 1 — The RNA-seq data with clinical information were obtained from TCGA-GBM and TCGA-LGG projects, and correlation of LYRM4-AS1 expression and gender of glioma patients was analyzed. [file peerj-11-16104-s001.jpg]

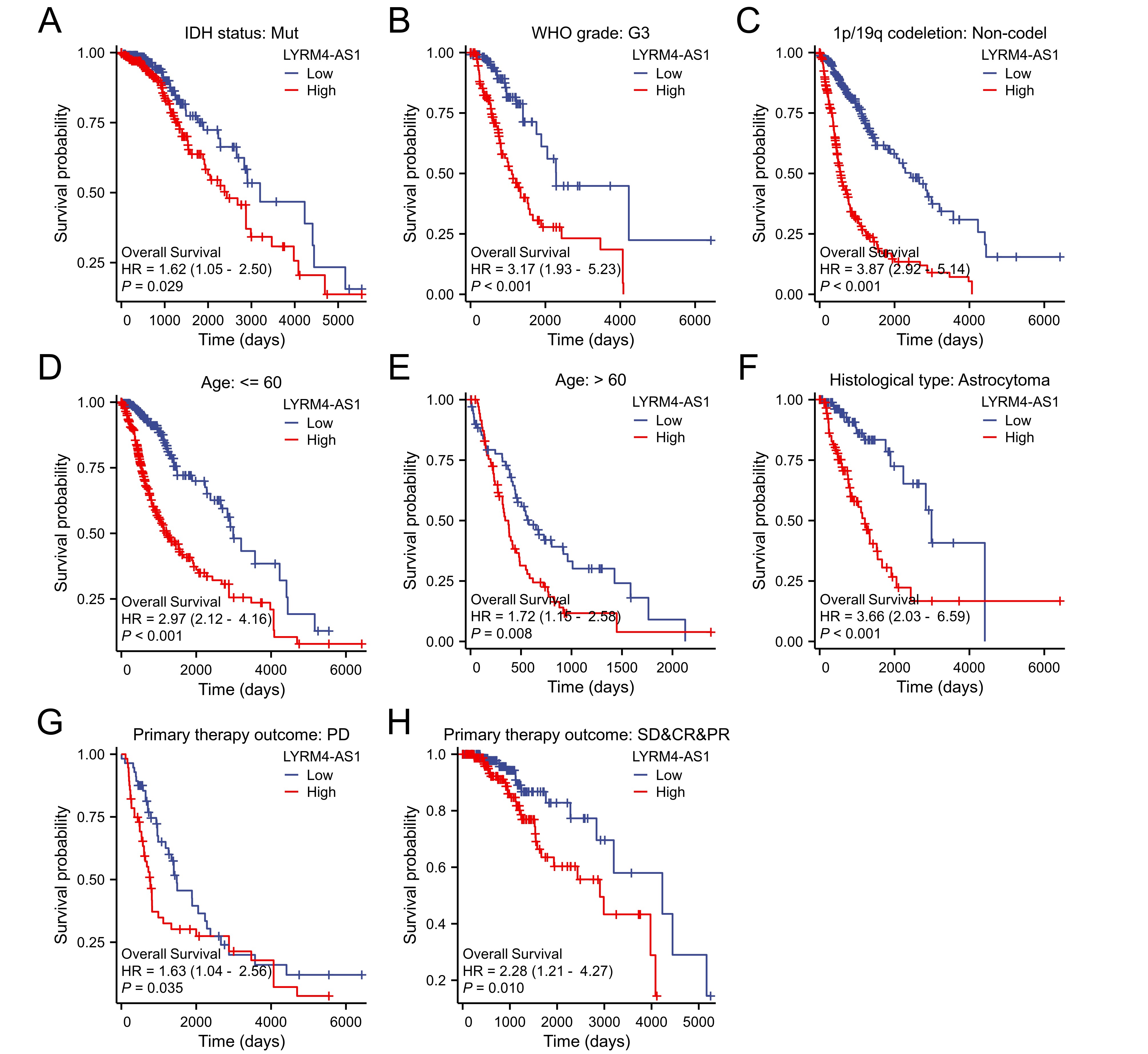

Supplement: Supplemental Information 2 — Prognostic analysis of high LYRM4-AS1 expression in IDH status:Mut (A), WHO grade: G3 (B), 1p/19q codeletion: Non-codel (C), Age: ≤60 (D, Age: > 60 (E), Histological type: Astrocytoma (F), Primary therapy outcome: PD (G), Primary therapy outcome: SD&CR&PR (H) of glioma patients. SD, stable disease; PR, partial response; CR, complete response; PD, progressive disease. The RNA-seq data with clinical information were obtained from TCGA-GBM and TCGA-LGG projects. [file peerj-11-16104-s002.jpg]

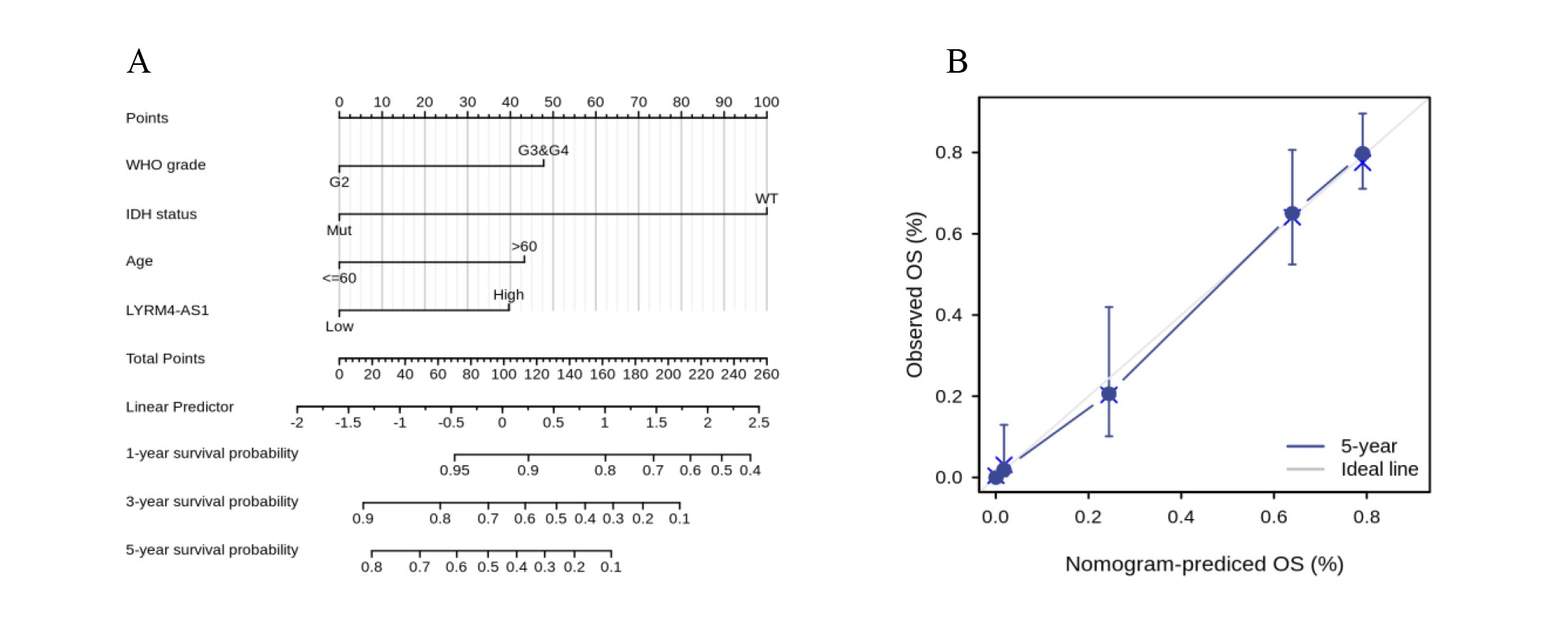

Supplement: Supplemental Information 3 — The OS nomogram based on WHO grade, IDH status, age and LYRM4-AS1 was constructed (A). The C-index of the nomograms was 0.843 (95% CI [0.832–0.854]). Drawing a vertical line from the total point axis straight downward to the outcome axis can pick up the probability of patients with glioma at 1,3,5-years. The calibration curve nomogram predicting 5-year OS (B). The horizontal coordinate was the survival probability predicted by the OS nomogram and the vertical coordinate is the actual survival probability. The 5-year survival predicted was indicated by the blue line and the gray line represented the ideal situation in which predicted and actual survival coincide. The closer the predicted line was to the diagonal line, the more accurate the model was. [file peerj-11-16104-s003.jpg]

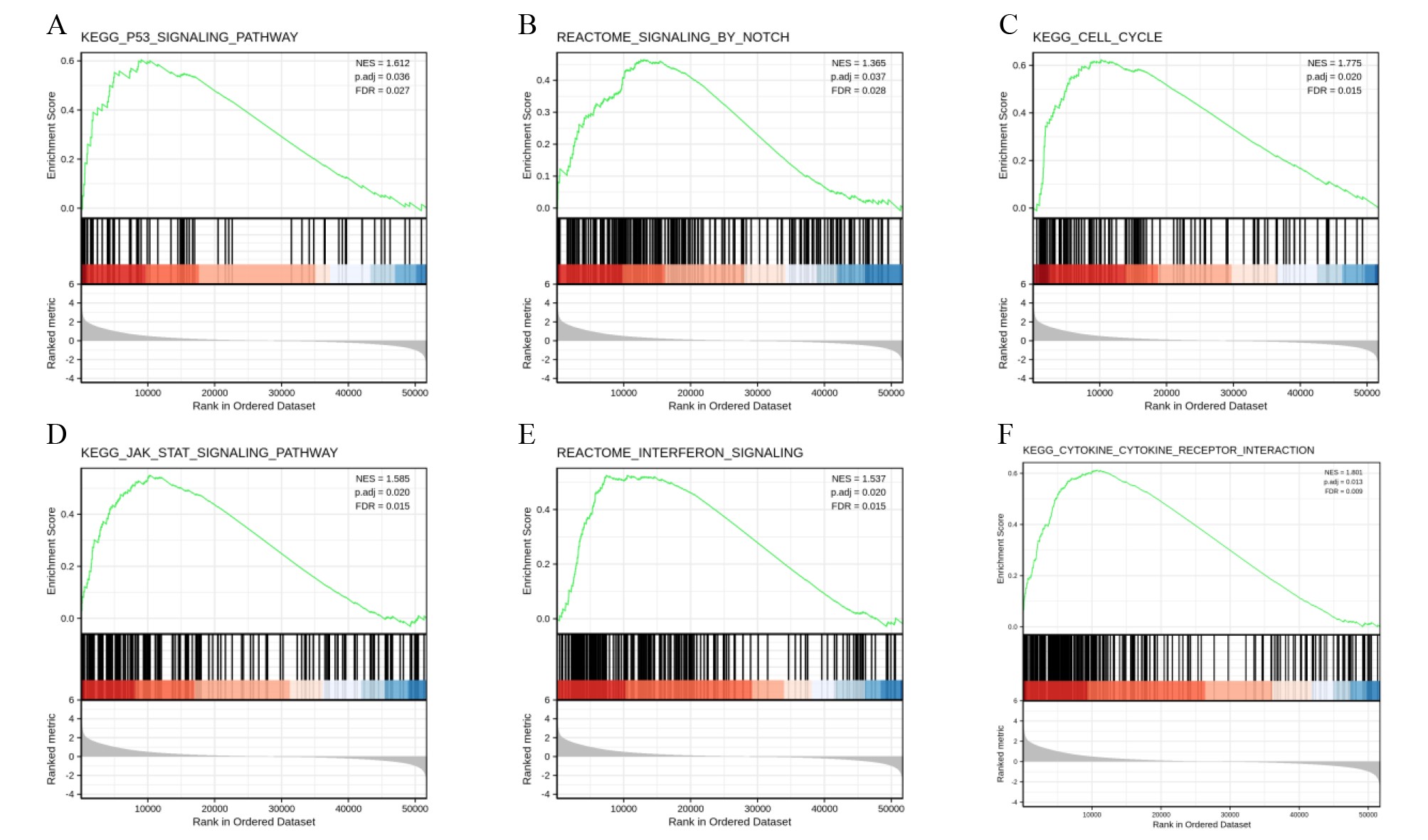

Supplement: Supplemental Information 4 — Pathways and biological processes were differentially enriched in LYRM4-AS1-related phenotype, including P53 signaling pathway (A), signaling by NOTCH (B), cell cycle (C), JAK_STAT signaling pathway (D), interferon signaling (E) and cytokine-cytokine receptor interaction (F). The top portion showed the enrichment scores. If the normalized enrichment score (NES) was positive, a peak appeared on the left side, indicating that the core molecules of the gene set were mainly concentrated in the high expression group on the left side. Each vertical line in the middle represented one molecule in the gene set. The lower part visualized the values after normalizing the gene set expression data. The RNA-seq data were obtained from TCGA-GBM and TCGA-LGG projects. [file peerj-11-16104-s004.jpg]

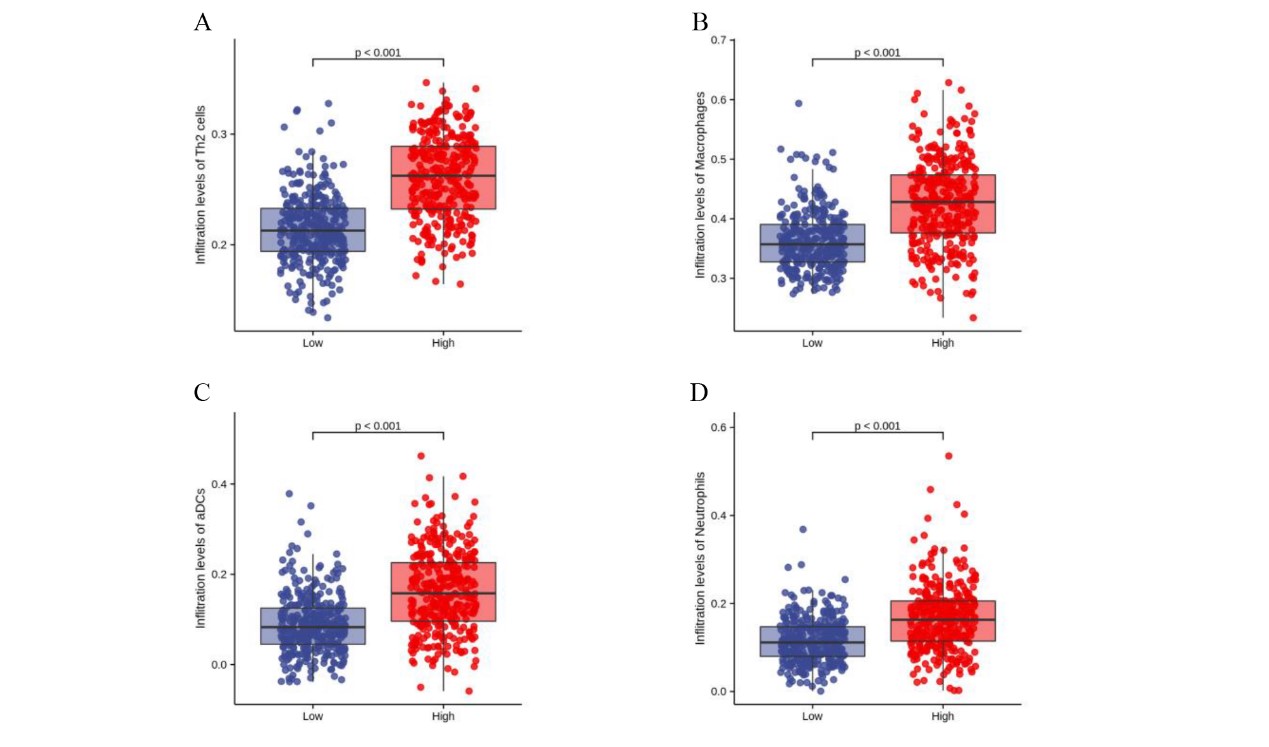

Supplement: Supplemental Information 5 — The analysis of LYRM4-AS1 expression levels and immune cell infiltration levels of Th2 cells (A), Macrophages (B), aDCs (C) and Neutrophils (D) compared with low LYRM4-AS1 expression groups. RNA-seq data of glioma patients were from GBM and LGG projects of TCGA. [file peerj-11-16104-s005.jpg]
