# Supplementary material for: High LYRM4-AS1 predicts poor prognosis in patients with glioma and correlates with immune infiltration
Source: PeerJ. 2023 Oct 3;11:e16104. doi: 10.7717/peerj.16104 (PMC10557942; doi:10.7717/peerj.16104)
Supplement: Supplemental Information 6 [file peerj-11-16104-s006.doc]

**Supplementary Table 1. The identified DEGs between high and low LYRM4-AS1 expression group of glioma tissues.**

| **gene_id** | **log2FoldChange** | **pvalue** | **padj** | **gene_name** | **gene_biotype** | **correlation** |
| --- | --- | --- | --- | --- | --- | --- |
| ENSG00000148848 | 3.147596364 | 0.0000 | 0.000000 | ADAM12 | protein_coding | 0.688257 |
| ENSG00000211829 | 4.043621985 | 0.0000 | 0.000000 | TRDC | TR_C_gene | 0.580632 |
| ENSG00000163661 | 3.3134403 | 0.0000 | 0.000000 | PTX3 | protein_coding | 0.664301 |
| ENSG00000255737 | 3.097671927 | 0.0000 | 0.000000 | AGAP2-AS1 | protein_coding | 0.510955 |
| ENSG00000134339 | 5.178483997 | 0.0000 | 0.000000 | SAA2 | protein_coding | 0.465784 |
| ENSG00000128342 | 3.256346547 | 0.0000 | 0.000000 | LIF | protein_coding | 0.597337 |
| ENSG00000154997 | 4.817276697 | 0.0000 | 0.000000 | 44088 | protein_coding | 0.476662 |
| ENSG00000164879 | 3.56771022 | 0.0000 | 0.000000 | CA3 | protein_coding | 0.573633 |
| ENSG00000269155 | 3.337741889 | 0.0000 | 0.000000 | AL009178.1 | protein_coding | 0.60575 |
| ENSG00000130600 | 4.592938448 | 0.0000 | 0.000000 | H19 | processed_transcript | 0.454293 |
| ENSG00000149948 | 4.283298957 | 0.0000 | 0.000000 | HMGA2 | protein_coding | 0.536954 |
| ENSG00000122176 | 3.313101201 | 0.0000 | 0.000000 | FMOD | protein_coding | 0.524867 |
| ENSG00000168542 | 3.248979976 | 0.0000 | 0.000000 | COL3A1 | protein_coding | 0.601596 |
| ENSG00000173432 | 5.505954314 | 0.0000 | 0.000000 | SAA1 | protein_coding | 0.503887 |
| ENSG00000164093 | 4.493702458 | 0.0000 | 0.000000 | PITX2 | protein_coding | 0.445263 |
| ENSG00000077274 | 3.714948948 | 0.0000 | 0.000000 | CAPN6 | protein_coding | 0.491225 |
| ENSG00000108821 | 3.230307509 | 0.0000 | 0.000000 | COL1A1 | protein_coding | 0.55643 |
| ENSG00000166741 | 3.285353982 | 0.0000 | 0.000000 | NNMT | protein_coding | 0.494755 |
| ENSG00000169245 | 3.219914453 | 0.0000 | 0.000000 | CXCL10 | protein_coding | 0.595353 |
| ENSG00000125726 | 3.516151081 | 0.0000 | 0.000000 | CD70 | protein_coding | 0.493333 |
| ENSG00000251593 | 3.063423462 | 0.0000 | 0.000000 | MSNP1 | pseudogene | 0.587944 |
| ENSG00000133110 | 4.410370797 | 0.0000 | 0.000000 | POSTN | protein_coding | 0.490912 |
| ENSG00000163359 | 3.035930806 | 0.0000 | 0.000000 | COL6A3 | protein_coding | 0.459873 |
| ENSG00000204277 | 3.27640433 | 0.0000 | 0.000000 | RP11-219G17.4 | lincRNA | 0.523337 |
| ENSG00000250526 | 3.191767169 | 0.0000 | 0.000000 | CCT6P2 | pseudogene | 0.511383 |
| ENSG00000136542 | 3.627951207 | 0.0000 | 0.000000 | GALNT5 | protein_coding | 0.576016 |
| ENSG00000131203 | 3.685274601 | 0.0000 | 0.000000 | IDO1 | protein_coding | 0.540359 |
| ENSG00000136231 | 3.095005632 | 0.0000 | 0.000000 | IGF2BP3 | protein_coding | 0.619473 |
| ENSG00000124731 | 3.00589708 | 0.0000 | 0.000000 | TREM1 | protein_coding | 0.535751 |
| ENSG00000172061 | 3.289035166 | 0.0000 | 0.000000 | LRRC15 | protein_coding | 0.501665 |
| ENSG00000029559 | 4.324313879 | 0.0000 | 0.000000 | IBSP | protein_coding | 0.496335 |
| ENSG00000237819 | 3.183466434 | 0.0000 | 0.000000 | AC002454.1 | antisense | 0.442369 |
| ENSG00000253676 | 3.098993492 | 0.0000 | 0.000000 | TAGLN2P1 | pseudogene | 0.557205 |
| ENSG00000188257 | 5.183416676 | 0.0000 | 0.000000 | PLA2G2A | protein_coding | 0.513445 |
| ENSG00000120093 | 3.254223932 | 0.0000 | 0.000000 | HOXB3 | protein_coding | 0.517549 |
| ENSG00000170454 | 3.84315292 | 0.0000 | 0.000000 | KRT75 | protein_coding | 0.44189 |
| ENSG00000124102 | 4.047072266 | 0.0000 | 0.000000 | PI3 | protein_coding | 0.37504 |
| ENSG00000170162 | 3.786326343 | 0.0000 | 0.000000 | VGLL2 | protein_coding | 0.479928 |
| ENSG00000100985 | 3.23625322 | 0.0000 | 0.000000 | MMP9 | protein_coding | 0.592052 |
| ENSG00000168447 | 3.093008098 | 0.0000 | 0.000000 | SCNN1B | protein_coding | 0.533153 |
| ENSG00000257017 | 3.479580213 | 0.0000 | 0.000000 | HP | protein_coding | 0.46976 |
| ENSG00000112238 | 4.086459024 | 0.0000 | 0.000000 | PRDM13 | protein_coding | 0.391804 |
| ENSG00000159217 | 3.263614979 | 0.0000 | 0.000000 | IGF2BP1 | protein_coding | 0.47551 |
| ENSG00000230838 | 3.428677614 | 0.0000 | 0.000000 | AC093850.2 | lincRNA | 0.534651 |
| ENSG00000133048 | 3.416475025 | 0.0000 | 0.000000 | CHI3L1 | protein_coding | 0.531397 |
| ENSG00000135903 | 4.297331207 | 0.0000 | 0.000000 | PAX3 | protein_coding | 0.503552 |
| ENSG00000183671 | 3.170694013 | 0.0000 | 0.000000 | GPR1 | protein_coding | 0.49335 |
| ENSG00000106511 | 3.14806922 | 0.0000 | 0.000000 | MEOX2 | protein_coding | 0.513979 |
| ENSG00000253187 | 5.032529586 | 0.0000 | 0.000000 | HOXA10-AS | antisense | 0.478463 |
| ENSG00000168779 | 3.168346662 | 0.0000 | 0.000000 | SHOX2 | protein_coding | 0.614974 |
| ENSG00000249001 | 3.986456699 | 0.0000 | 0.000000 | RP11-742B18.1 | antisense | 0.511604 |
| ENSG00000012223 | 3.588675291 | 0.0000 | 0.000000 | LTF | protein_coding | 0.532536 |
| ENSG00000078399 | 3.812142756 | 0.0000 | 0.000000 | HOXA9 | protein_coding | 0.495186 |
| ENSG00000234964 | 3.202374884 | 0.0000 | 0.000000 | FABP5P7 | pseudogene | 0.48534 |
| ENSG00000128710 | 3.486765425 | 0.0000 | 0.000000 | HOXD10 | protein_coding | 0.541275 |
| ENSG00000197587 | 3.215020649 | 0.0000 | 0.000000 | DMBX1 | protein_coding | 0.502774 |
| ENSG00000237987 | 3.066538498 | 0.0000 | 0.000000 | RP11-503C24.2 | lincRNA | 0.50344 |
| ENSG00000164326 | -3.578251333 | 0.0000 | 0.000000 | CARTPT | protein_coding | -0.19694 |
| ENSG00000241749 | 3.041853974 | 0.0000 | 0.000000 | RPSAP52 | pseudogene | 0.476362 |
| ENSG00000270168 | 3.358446115 | 0.0000 | 0.000000 | LA16c-380H5.4 | lincRNA | 0.205539 |
| ENSG00000137745 | 4.11223104 | 0.0000 | 0.000000 | MMP13 | protein_coding | 0.357401 |
| ENSG00000236969 | 3.112148512 | 0.0000 | 0.000000 | GGT8P | pseudogene | 0.42116 |
| ENSG00000223823 | 3.680252796 | 0.0000 | 0.000000 | RP11-465B22.5 | lincRNA | 0.509235 |
| ENSG00000204362 | 3.877157293 | 0.0000 | 0.000000 | RP11-380J14.1 | lincRNA | 0.467334 |
| ENSG00000186973 | 3.067094632 | 0.0000 | 0.000000 | FAM183A | protein_coding | 0.47012 |
| ENSG00000186191 | 3.111302447 | 0.0000 | 0.000000 | BPIFB4 | protein_coding | 0.390005 |
| ENSG00000181541 | 3.014475065 | 0.0000 | 0.000000 | MAB21L2 | protein_coding | 0.283056 |
| ENSG00000246095 | 3.583012477 | 0.0000 | 0.000000 | LINC01096 | lincRNA | 0.467307 |
| ENSG00000228630 | 4.41938486 | 0.0000 | 0.000000 | HOTAIR | antisense | 0.567707 |
| ENSG00000253552 | 3.121584011 | 0.0000 | 0.000000 | HOXA-AS2 | antisense | 0.557437 |
| ENSG00000229637 | 4.217199406 | 0.0000 | 0.000000 | PRAC2 | antisense | 0.457185 |
| ENSG00000250133 | 3.506092349 | 0.0000 | 0.000000 | HOXC-AS2 | antisense | 0.562849 |
| ENSG00000223703 | 3.007944951 | 0.0000 | 0.000000 | AC027612.4 | pseudogene | 0.521137 |
| ENSG00000184937 | 3.163527663 | 0.0000 | 0.000000 | WT1 | protein_coding | 0.303743 |
| ENSG00000171956 | 3.284464739 | 0.0000 | 0.000000 | FOXB1 | protein_coding | 0.423332 |
| ENSG00000255071 | 5.525729235 | 0.0000 | 0.000000 | SAA2-SAA4 | processed_transcript | 0.437597 |
| ENSG00000183242 | 3.626994535 | 0.0000 | 0.000000 | WT1-AS | antisense | 0.360154 |
| ENSG00000236972 | 3.057485042 | 0.0000 | 0.000000 | FABP5P1 | pseudogene | 0.479747 |
| ENSG00000128714 | 3.723403639 | 0.0000 | 0.000000 | HOXD13 | protein_coding | 0.56449 |
| ENSG00000159184 | 3.450248657 | 0.0000 | 0.000000 | HOXB13 | protein_coding | 0.470918 |
| ENSG00000170689 | 3.068344851 | 0.0000 | 0.000000 | HOXB9 | protein_coding | 0.515827 |
| ENSG00000120068 | 3.739015898 | 0.0000 | 0.000000 | HOXB8 | protein_coding | 0.464727 |
| ENSG00000136574 | 3.002673646 | 0.0000 | 0.000000 | GATA4 | protein_coding | 0.504435 |
| ENSG00000108688 | 3.779144563 | 0.0000 | 0.000000 | CCL7 | protein_coding | 0.282195 |
| ENSG00000128713 | 3.703498463 | 0.0000 | 0.000000 | HOXD11 | protein_coding | 0.560882 |
| ENSG00000112077 | 3.051741253 | 0.0000 | 0.000000 | RHAG | protein_coding | 0.472755 |
| ENSG00000249641 | 4.350988456 | 0.0000 | 0.000000 | HOXC13-AS | antisense | 0.497329 |
| ENSG00000233123 | -3.441081873 | 0.0000 | 0.000000 | LINC01007 | lincRNA | -0.32239 |
| ENSG00000125798 | 3.201231721 | 0.0000 | 0.000000 | FOXA2 | protein_coding | 0.294846 |
| ENSG00000236453 | 3.73149683 | 0.0000 | 0.000000 | AC003092.1 | lincRNA | 0.430628 |
| ENSG00000118113 | 3.028641288 | 0.0000 | 0.000000 | MMP8 | protein_coding | 0.346251 |
| ENSG00000123364 | 3.820205828 | 0.0000 | 0.000000 | HOXC13 | protein_coding | 0.551378 |
| ENSG00000236663 | 3.216197714 | 0.0000 | 0.000000 | AP001631.9 | antisense | 0.468976 |
| ENSG00000037965 | 3.060024149 | 0.0000 | 0.000000 | HOXC8 | protein_coding | 0.531912 |
| ENSG00000233730 | 3.118273548 | 0.0000 | 0.000000 | RP4-666F24.3 | lincRNA | 0.362437 |
| ENSG00000173467 | 3.105009055 | 0.0000 | 0.000000 | AGR3 | protein_coding | 0.403163 |
| ENSG00000251587 | 3.05399612 | 0.0000 | 0.000000 | LDHAP1 | pseudogene | 0.488492 |
| ENSG00000213201 | 3.015612382 | 0.0000 | 0.000000 | FABP5P10 | pseudogene | 0.44508 |
| ENSG00000225107 | 3.705120387 | 0.0000 | 0.000000 | AC092484.1 | lincRNA | 0.288537 |
| ENSG00000106006 | 3.23605076 | 0.0000 | 0.000000 | HOXA6 | protein_coding | 0.607069 |
| ENSG00000163081 | 3.90438348 | 0.0000 | 0.000000 | CCDC140 | protein_coding | 0.514934 |
| ENSG00000123388 | 3.536294155 | 0.0000 | 0.000000 | HOXC11 | protein_coding | 0.534425 |
| ENSG00000227706 | 3.29466345 | 0.0000 | 0.000000 | RP11-301G19.1 | lincRNA | 0.457772 |
| ENSG00000231817 | 3.183827639 | 0.0000 | 0.000000 | RP11-189B4.6 | lincRNA | 0.503632 |
| ENSG00000202111 | 3.738216337 | 0.0000 | 0.000000 | VTRNA1-2 | misc_RNA | 0.334412 |
| ENSG00000232835 | 3.840727533 | 0.0000 | 0.000000 | AC107057.1 | lincRNA | 0.29016 |
| ENSG00000178776 | 3.489986286 | 0.0000 | 0.000000 | C5orf46 | protein_coding | 0.364652 |
| ENSG00000204542 | 3.909470818 | 0.0000 | 0.000000 | C6orf15 | protein_coding | 0.474839 |
| ENSG00000228742 | 3.558316441 | 0.0000 | 0.000000 | RP5-884M6.1 | lincRNA | 0.356868 |
| ENSG00000163646 | 3.261590291 | 0.0000 | 0.000000 | CLRN1 | protein_coding | 0.299277 |
| ENSG00000112246 | 3.054568894 | 0.0000 | 0.000000 | SIM1 | protein_coding | 0.285173 |
| ENSG00000231317 | 3.16981309 | 0.0000 | 0.000000 | RP11-310H4.6 | pseudogene | 0.328625 |
| ENSG00000248749 | 3.291586527 | 0.0000 | 0.000000 | RP11-42A4.1 | lincRNA | 0.442224 |
| ENSG00000180483 | -3.98791776 | 0.0000 | 0.000000 | DEFB119 | protein_coding | -0.33639 |
| ENSG00000253821 | 3.013795629 | 0.0000 | 0.000000 | RP11-246K15.1 | lincRNA | 0.435361 |
| ENSG00000250451 | 3.036012603 | 0.0000 | 0.000000 | HOXC-AS1 | antisense | 0.538849 |
| ENSG00000259974 | 3.122216684 | 0.0000 | 0.000000 | LINC00261 | lincRNA | 0.315876 |
| ENSG00000136696 | 3.193675051 | 0.0000 | 0.000000 | IL36B | protein_coding | 0.404784 |
| ENSG00000242407 | 3.137890936 | 0.0000 | 0.000000 | CTD-2377D24.4 | lincRNA | 0.258799 |
