# Supplementary material for: High LYRM4-AS1 predicts poor prognosis in patients with glioma and correlates with immune infiltration
Source: PeerJ. 2023 Oct 3;11:e16104. doi: 10.7717/peerj.16104 (PMC10557942; doi:10.7717/peerj.16104)
Supplement: Supplemental Information 7 [file peerj-11-16104-s007.doc]

**Supplementary Table 2. The results of the GO enrichment analysis.**

| **ONTOLOGY** | **ID** | **Description** | **GeneRatio** | **BgRatio** | **pvalue** | **p.adjust** | **qvalue** | **geneID** | **Count** |
| --- | --- | --- | --- | --- | --- | --- | --- | --- | --- |
| BP | GO:0007389 | pattern specification process | 51/363 | 446/18670 | 0.00 | 0.00 | 0.00 | MEOX2/CELSR1/HOXB3/DMRTA2/STC1/HOXA10/HOXC10/HOXB2/HOXA5/EN1/DKK1/HOXD9/HOXD10/SP8/HOXB7/HOXA3/HOXD13/NKX2-5/HOXB4/DNAH11/GSX2/HAND2/HOXA2/HOXA4/HOXA7/HOXA9/HOXD11/HOXC6/HOXC13/HOXC11/FEZF1/HOXC9/NKX3-2/PITX2/GATA4/HOXC8/HOXB8/HOXA6/WT1/HOXB5/BARX1/HOXB9/LBX1/FOXB1/FOXA2/RIPPLY3/CER1/HOXD12/HOXC5/TBX20/GDF3 | 51 |
| BP | GO:0009952 | anterior/posterior pattern specification | 37/363 | 219/18670 | 0.00 | 0.00 | 0.00 | MEOX2/CELSR1/HOXB3/HOXA10/HOXC10/HOXB2/HOXA5/EN1/DKK1/HOXD9/HOXD10/HOXB7/HOXA3/HOXD13/HOXB4/HOXA2/HOXA4/HOXA7/HOXA9/HOXC6/HOXC13/HOXC11/FEZF1/HOXC9/GATA4/HOXC8/HOXB8/HOXA6/WT1/HOXB5/BARX1/HOXB9/FOXB1/FOXA2/CER1/HOXC5/GDF3 | 37 |
| BP | GO:0003002 | regionalization | 44/363 | 351/18670 | 0.00 | 0.00 | 0.00 | MEOX2/CELSR1/HOXB3/DMRTA2/HOXA10/HOXC10/HOXB2/HOXA5/EN1/DKK1/HOXD9/HOXD10/SP8/HOXB7/HOXA3/HOXD13/NKX2-5/HOXB4/GSX2/HOXA2/HOXA4/HOXA7/HOXA9/HOXD11/HOXC6/HOXC13/HOXC11/FEZF1/HOXC9/PITX2/GATA4/HOXC8/HOXB8/HOXA6/WT1/HOXB5/BARX1/HOXB9/FOXB1/FOXA2/CER1/HOXC5/TBX20/GDF3 | 44 |
| BP | GO:0048706 | embryonic skeletal system development | 24/363 | 126/18670 | 0.00 | 0.00 | 0.00 | COL1A1/HOXB3/SHOX2/HOXB2/HOXA5/HOXD9/HOXD10/HOXB7/HOXA3/HOXB4/HOXA2/HOXA4/HOXA7/HOXA9/HOXC6/HOXC11/HOXC9/HOXA1/NKX3-2/HOXB8/HOXA6/HOXB5/HOXB9/HOXC5 | 24 |
| BP | GO:0048568 | embryonic organ development | 41/363 | 428/18670 | 0.00 | 0.00 | 0.00 | VEGFA/COL5A2/RARRES2/CXCL8/LIF/CELSR1/CTHRC1/HOXB3/SHOX2/E2F7/MFAP2/HOXB2/CHRNA9/HOXA5/EN1/HOXD9/HOXD10/HOXB7/HOXA3/NKX2-5/HOXB4/HAND2/HOXA2/HOXA4/HOXA7/WNT16/PHLDA2/HOXC11/HOXC9/HOXA1/NKX3-2/GATA4/HOXB8/HOXA6/HOXB5/LBX1/CLRN1/TBX20/HMX3/GDF3/CSF2 | 41 |
| BP | GO:0048705 | skeletal system morphogenesis | 30/363 | 239/18670 | 0.00 | 0.00 | 0.00 | LTF/COL1A1/ANXA2/COL6A2/COL6A3/HOXB3/SHOX2/STC1/HOXB2/HOXA5/WNT10B/HOXD9/HOXD10/HOXB7/HOXA3/HOXB4/HOXA2/HOXA4/HOXA7/HOXC11/HOXC9/HOXA1/NKX3-2/HOXC8/HOXB8/HOXA6/HOXB5/MMP13/CER1/OTOR | 30 |
| BP | GO:0030198 | extracellular matrix organization | 36/363 | 368/18670 | 0.00 | 0.00 | 0.00 | COL4A2/COL1A2/COL4A1/TIMP1/COL1A1/ANXA2/COL3A1/COL6A2/PDPN/TGFBI/SERPINE1/POSTN/COL5A2/FMOD/THBS1/COL5A1/COL6A3/LOX/COL8A1/MMP9/LUM/ADAM12/PTX3/LOXL1/IBSP/MMP19/TNFRSF11B/MFAP2/DPP4/MMP11/MMP7/KLK7/WT1/MMP13/KLK5/MMP8 | 36 |
| BP | GO:0043062 | extracellular structure organization | 38/363 | 422/18670 | 0.00 | 0.00 | 0.00 | COL4A2/COL1A2/COL4A1/TIMP1/COL1A1/ANXA2/COL3A1/COL6A2/PDPN/TGFBI/SERPINE1/POSTN/COL5A2/FMOD/THBS1/COL5A1/COL6A3/LOX/COL8A1/PLA2G2A/MMP9/LUM/ADAM12/PTX3/SDC1/LOXL1/IBSP/MMP19/TNFRSF11B/MFAP2/DPP4/MMP11/MMP7/KLK7/WT1/MMP13/KLK5/MMP8 | 38 |
| BP | GO:0048562 | embryonic organ morphogenesis | 31/363 | 288/18670 | 0.00 | 0.00 | 0.00 | CELSR1/CTHRC1/HOXB3/SHOX2/MFAP2/HOXB2/CHRNA9/HOXA5/HOXD9/HOXD10/HOXB7/HOXA3/NKX2-5/HOXB4/HAND2/HOXA2/HOXA4/HOXA7/WNT16/HOXC11/HOXC9/HOXA1/NKX3-2/GATA4/HOXB8/HOXA6/HOXB5/LBX1/CLRN1/TBX20/HMX3 | 31 |
| BP | GO:0048704 | embryonic skeletal system morphogenesis | 18/363 | 93/18670 | 0.00 | 0.00 | 0.00 | HOXB3/SHOX2/HOXB2/HOXA5/HOXD9/HOXD10/HOXB7/HOXA3/HOXB4/HOXA2/HOXA4/HOXA7/HOXC11/HOXC9/HOXA1/HOXB8/HOXA6/HOXB5 | 18 |
| CC | GO:0062023 | collagen-containing extracellular matrix | 34/372 | 406/19717 | 0.00 | 0.00 | 0.00 | COL4A2/COL1A2/COL4A1/TIMP1/COL1A1/ANXA2/COL3A1/ANXA1/COL6A2/TGFBI/SERPINE1/POSTN/COL5A2/F13A1/FMOD/THBS1/COL5A1/PCOLCE/COL6A3/RARRES2/S100A9/MXRA5/COL8A1/MMP9/LUM/SRPX2/S100A4/GDF10/LOXL1/CTHRC1/GDF15/MFAP2/LRRC15/MMP8 | 34 |
| MF | GO:0005201 | extracellular matrix structural constituent | 22/351 | 163/17697 | 0.00 | 0.00 | 0.00 | CHI3L1/COL4A2/COL1A2/COL4A1/COL1A1/COL3A1/COL6A2/TGFBI/POSTN/COL5A2/FMOD/THBS1/COL5A1/PCOLCE/COL6A3/MXRA5/COL8A1/LUM/SRPX2/CTHRC1/MFAP2/TFPI2 | 22 |
| CC | GO:0098644 | complex of collagen trimers | 9/372 | 19/19717 | 0.00 | 0.00 | 0.00 | COL4A2/COL1A2/COL4A1/COL1A1/COL3A1/COL5A2/COL5A1/COL8A1/LUM | 9 |
| BP | GO:0051216 | cartilage development | 22/363 | 209/18670 | 0.00 | 0.00 | 0.00 | CHI3L1/TIMP1/COL1A1/ANXA2/COL6A2/TGFBI/COL6A3/LUM/HOXB3/SHOX2/STC1/CCN4/HOXA5/WNT10B/PITX1/HOXA3/HAND2/HMGA2/NKX3-2/MMP13/CER1/OTOR | 22 |
| BP | GO:0061448 | connective tissue development | 25/363 | 273/18670 | 0.00 | 0.00 | 0.00 | CHI3L1/TIMP1/COL1A1/ANXA2/COL6A2/TGFBI/COL5A1/COL6A3/LOX/LUM/HOXB3/SHOX2/STC1/CCN4/HOXA5/WNT10B/PITX1/HOXA3/HAND2/HMGA2/NKX3-2/WT1/MMP13/CER1/OTOR | 25 |
| MF | GO:0001228 | DNA-binding transcription activator activity, RNA polymerase II-specific | 32/351 | 439/17697 | 0.00 | 0.00 | 0.00 | MYBL2/MEOX2/HOXB3/HOXA10/HOXC10/HOXB2/HOXA5/NEUROD6/HOXD10/PITX1/HOXB7/HOXD13/NKX2-5/HOXB4/SOHLH1/HAND2/HOXA4/HOXA7/HMGA2/HOXC13/HOXC11/SIX6/HOXA1/TFAP2B/GATA4/WT1/HOXB5/BARX1/ELF5/CREB3L3/HNF4A/TBX20 | 32 |
| BP | GO:0019730 | antimicrobial humoral response | 17/363 | 122/18670 | 0.00 | 0.00 | 0.00 | LTF/RARRES2/LYZ/S100A9/CXCL8/PLA2G2A/CXCL10/PI3/CXCL9/CXCL11/KLK7/CXCL6/KLK5/KRT6A/CAMP/REG1B/H2BS1 | 17 |
| MF | GO:0048018 | receptor ligand activity | 33/351 | 482/17697 | 0.00 | 0.00 | 0.00 | NAMPT/TIMP1/VEGFA/CXCL8/CCK/SAA1/LIF/CXCL10/GDF10/GDF15/STC1/TNFRSF11B/CLCF1/WNT10B/CXCL9/DKK1/CXCL11/SAA2/CARTPT/GAL/TNFSF14/CCL20/CXCL6/CD70/VSTM1/CCL7/RETN/CER1/IL36B/FGF3/FGF19/GDF3/CSF2 | 33 |
| MF | GO:0005125 | cytokine activity | 22/351 | 220/17697 | 0.00 | 0.00 | 0.00 | NAMPT/TIMP1/VEGFA/CXCL8/LIF/CXCL10/GDF10/GDF15/TNFRSF11B/CLCF1/CXCL9/CXCL11/TNFSF14/CCL20/CXCL6/CD70/VSTM1/CCL7/CER1/IL36B/GDF3/CSF2 | 22 |
| CC | GO:0005581 | collagen trimer | 14/372 | 87/19717 | 0.00 | 0.00 | 0.00 | COL4A2/COL1A2/COL4A1/COL1A1/COL3A1/COL6A2/COL5A2/COL5A1/COL6A3/LOX/COL8A1/LUM/CTHRC1/MARCO | 14 |
| BP | GO:0043588 | skin development | 30/363 | 419/18670 | 0.00 | 0.00 | 0.00 | COL1A2/COL1A1/COL3A1/ANXA1/COL5A2/COL5A1/CELSR1/PI3/WNT10B/CYP27B1/DKK1/DSG2/VDR/HOXA7/WNT16/GAL/HOXC13/TFAP2B/KRT75/KRT80/KRT14/KLK5/KRT6A/KRT31/SPRR1B/LIPN/SPRR3/KRT33B/KRT6C/SPRR2E | 30 |
| BP | GO:0061844 | antimicrobial humoral immune response mediated by antimicrobial peptide | 13/363 | 73/18670 | 0.00 | 0.00 | 0.00 | LTF/S100A9/CXCL8/CXCL10/CXCL9/CXCL11/KLK7/CXCL6/KLK5/KRT6A/CAMP/REG1B/H2BS1 | 13 |
| MF | GO:0030020 | extracellular matrix structural constituent conferring tensile strength | 10/351 | 41/17697 | 0.00 | 0.00 | 0.00 | COL4A2/COL1A2/COL4A1/COL1A1/COL3A1/COL6A2/COL5A2/COL5A1/COL6A3/COL8A1 | 10 |
| BP | GO:0008544 | epidermis development | 31/363 | 464/18670 | 0.00 | 0.00 | 0.00 | ANXA1/FABP5/CELSR1/PI3/WNT10B/CYP27B1/DKK1/DSG2/KLK7/VDR/HOXA7/WNT16/HOXB13/GAL/HOXC13/PITX2/KRT75/KRT80/KRT14/KLK5/KRT6A/KRT31/SPRR1B/CLRN1/LIPN/SPRR3/KRT33B/KRT6C/GDF3/CALML5/SPRR2E | 31 |
| CC | GO:0005583 | fibrillar collagen trimer | 6/372 | 11/19717 | 0.00 | 0.00 | 0.00 | COL1A2/COL1A1/COL3A1/COL5A2/COL5A1/LUM | 6 |
| CC | GO:0098643 | banded collagen fibril | 6/372 | 11/19717 | 0.00 | 0.00 | 0.00 | COL1A2/COL1A1/COL3A1/COL5A2/COL5A1/LUM | 6 |
| CC | GO:0044420 | extracellular matrix component | 10/372 | 51/19717 | 0.00 | 0.00 | 0.00 | COL4A2/COL1A2/COL4A1/COL1A1/COL3A1/COL5A2/COL5A1/COL8A1/LUM/MFAP2 | 10 |
| BP | GO:0060173 | limb development | 18/363 | 179/18670 | 0.00 | 0.00 | 0.00 | MEOX2/SHOX2/HOXA10/HOXC10/EN1/DKK1/HOXD9/HOXD10/PITX1/SP8/HOXD13/HAND2/HOXA9/HOXC13/HOXC11/TFAP2B/PITX2/HOXD12 | 18 |
| BP | GO:0048736 | appendage development | 18/363 | 179/18670 | 0.00 | 0.00 | 0.00 | MEOX2/SHOX2/HOXA10/HOXC10/EN1/DKK1/HOXD9/HOXD10/PITX1/SP8/HOXD13/HAND2/HOXA9/HOXC13/HOXC11/TFAP2B/PITX2/HOXD12 | 18 |
| BP | GO:0007492 | endoderm development | 12/363 | 76/18670 | 0.00 | 0.00 | 0.00 | COL4A2/COL5A2/COL5A1/COL8A1/MMP9/DKK1/HMGA2/HOXC11/GATA4/MMP8/TBX20/GDF3 | 12 |
| BP | GO:0070268 | cornification | 14/363 | 112/18670 | 0.00 | 0.00 | 0.00 | PI3/DSG2/KRT75/KRT80/KRT14/KLK5/KRT6A/KRT31/SPRR1B/LIPN/SPRR3/KRT33B/KRT6C/SPRR2E | 14 |
| BP | GO:0030199 | collagen fibril organization | 10/363 | 54/18670 | 0.00 | 0.00 | 0.00 | COL1A2/COL1A1/ANXA2/COL3A1/COL5A2/FMOD/COL5A1/LOX/LUM/MMP11 | 10 |
| BP | GO:0009954 | proximal/distal pattern formation | 8/363 | 31/18670 | 0.00 | 0.00 | 0.00 | HOXA10/HOXC10/EN1/HOXD9/HOXD10/SP8/HOXA9/HOXC11 | 8 |
| BP | GO:0030326 | embryonic limb morphogenesis | 14/363 | 125/18670 | 0.00 | 0.00 | 0.00 | SHOX2/HOXA10/HOXC10/EN1/DKK1/HOXD9/HOXD10/PITX1/SP8/HOXD13/HAND2/HOXA9/HOXC11/HOXD12 | 14 |
| BP | GO:0035113 | embryonic appendage morphogenesis | 14/363 | 125/18670 | 0.00 | 0.00 | 0.00 | SHOX2/HOXA10/HOXC10/EN1/DKK1/HOXD9/HOXD10/PITX1/SP8/HOXD13/HAND2/HOXA9/HOXC11/HOXD12 | 14 |
| BP | GO:0035108 | limb morphogenesis | 15/363 | 148/18670 | 0.00 | 0.00 | 0.00 | SHOX2/HOXA10/HOXC10/EN1/DKK1/HOXD9/HOXD10/PITX1/SP8/HOXD13/HAND2/HOXA9/HOXC11/TFAP2B/HOXD12 | 15 |
| BP | GO:0035107 | appendage morphogenesis | 15/363 | 148/18670 | 0.00 | 0.00 | 0.00 | SHOX2/HOXA10/HOXC10/EN1/DKK1/HOXD9/HOXD10/PITX1/SP8/HOXD13/HAND2/HOXA9/HOXC11/TFAP2B/HOXD12 | 15 |
| BP | GO:0022617 | extracellular matrix disassembly | 11/363 | 80/18670 | 0.00 | 0.00 | 0.00 | TIMP1/PDPN/MMP9/MMP19/DPP4/MMP11/MMP7/KLK7/MMP13/KLK5/MMP8 | 11 |
| BP | GO:0001706 | endoderm formation | 9/363 | 50/18670 | 0.00 | 0.00 | 0.00 | COL4A2/COL5A2/COL5A1/COL8A1/MMP9/DKK1/HMGA2/MMP8/TBX20 | 9 |
| MF | GO:0048407 | platelet-derived growth factor binding | 5/351 | 11/17697 | 0.00 | 0.00 | 0.00 | COL1A2/COL4A1/COL1A1/COL3A1/COL5A1 | 5 |
| BP | GO:0071621 | granulocyte chemotaxis | 13/363 | 123/18670 | 0.00 | 0.00 | 0.00 | ANXA1/THBS1/RARRES2/S100A9/CXCL8/SAA1/CXCL10/CXCL9/CXCL11/CCL20/CXCL6/CCL7/IL36B | 13 |
| CC | GO:0070820 | tertiary granule | 14/372 | 164/19717 | 0.00 | 0.00 | 0.00 | LTF/PLAU/LYZ/MMP9/PTX3/HP/CLEC5A/TNFAIP6/FPR2/CLEC12A/MCEMP1/ADGRE3/CAMP/MMP8 | 14 |
| BP | GO:0007517 | muscle organ development | 24/363 | 410/18670 | 0.00 | 0.00 | 0.00 | COL3A1/COL6A3/LOX/MEOX2/LIF/CXCL10/DMRTA2/SHOX2/CHODL/CHRNA1/WNT10B/DKK1/HOXD9/HOXD10/PITX1/NKX2-5/TNNT2/PAX3/TCF23/WT1/VGLL2/LBX1/FGF3/TBX20 | 24 |
| BP | GO:0030595 | leukocyte chemotaxis | 17/363 | 224/18670 | 0.00 | 0.00 | 0.00 | VEGFA/ANXA1/SERPINE1/THBS1/RARRES2/S100A9/CXCL8/SAA1/CXCL10/CXCL9/CXCL11/TNFSF14/FPR2/CCL20/CXCL6/CCL7/IL36B | 17 |
| BP | GO:0009913 | epidermal cell differentiation | 22/363 | 358/18670 | 0.00 | 0.00 | 0.00 | ANXA1/PI3/CYP27B1/DSG2/VDR/HOXA7/WNT16/PITX2/KRT75/KRT80/KRT14/KLK5/KRT6A/KRT31/SPRR1B/CLRN1/LIPN/SPRR3/KRT33B/KRT6C/GDF3/SPRR2E | 22 |
| BP | GO:0035987 | endodermal cell differentiation | 8/363 | 45/18670 | 0.00 | 0.00 | 0.00 | COL4A2/COL5A2/COL5A1/COL8A1/MMP9/DKK1/HMGA2/MMP8 | 8 |
| BP | GO:0060326 | cell chemotaxis | 20/363 | 304/18670 | 0.00 | 0.00 | 0.00 | VEGFA/ANXA1/SERPINE1/THBS1/RARRES2/S100A9/CXCL8/LOX/SAA1/CXCL10/CXCL9/CXCL11/SAA2/TNFSF14/FPR2/CCL20/CXCL6/HOXB9/CCL7/IL36B | 20 |
| BP | GO:0060351 | cartilage development involved in endochondral bone morphogenesis | 8/363 | 47/18670 | 0.00 | 0.00 | 0.00 | COL1A1/ANXA2/COL6A2/COL6A3/SHOX2/STC1/MMP13/CER1 | 8 |
| CC | GO:1904724 | tertiary granule lumen | 8/372 | 55/19717 | 0.00 | 0.00 | 0.00 | LTF/LYZ/MMP9/PTX3/HP/TNFAIP6/CAMP/MMP8 | 8 |
| BP | GO:0097529 | myeloid leukocyte migration | 16/363 | 210/18670 | 0.00 | 0.00 | 0.00 | VEGFA/ANXA1/SERPINE1/THBS1/RARRES2/S100A9/CXCL8/SAA1/CXCL10/CXCL9/CXCL11/FPR2/CCL20/CXCL6/CCL7/IL36B | 16 |
| BP | GO:0060135 | maternal process involved in female pregnancy | 9/363 | 64/18670 | 0.00 | 0.00 | 0.00 | LIF/GJB2/STC1/CYP27B1/MMP7/DSG2/VDR/TCF23/HMX3 | 9 |
| BP | GO:0097530 | granulocyte migration | 13/363 | 141/18670 | 0.00 | 0.00 | 0.00 | ANXA1/THBS1/RARRES2/S100A9/CXCL8/SAA1/CXCL10/CXCL9/CXCL11/CCL20/CXCL6/CCL7/IL36B | 13 |
| CC | GO:0042581 | specific granule | 13/372 | 160/19717 | 0.00 | 0.00 | 0.00 | CHI3L1/LTF/PLAU/LYZ/PTX3/HP/CLEC5A/FPR2/CLEC12A/MCEMP1/RETN/CAMP/MMP8 | 13 |
| BP | GO:0046697 | decidualization | 6/363 | 24/18670 | 0.00 | 0.00 | 0.00 | LIF/GJB2/STC1/CYP27B1/VDR/TCF23 | 6 |
| BP | GO:0007565 | female pregnancy | 15/363 | 192/18670 | 0.00 | 0.00 | 0.00 | IGFBP2/NAMPT/TIMP1/MMP9/LIF/GJB2/STC1/CYP27B1/MMP7/DSG2/VDR/IDO1/TCF23/EPYC/HMX3 | 15 |
| BP | GO:0045165 | cell fate commitment | 18/363 | 270/18670 | 0.00 | 0.00 | 0.00 | PDPN/DMRTA2/HOXC10/WNT10B/DKK1/HOXD10/PITX1/NKX2-5/GSX2/HOXA2/WNT16/WT1/LBX1/FOXA2/ELF5/TBX20/GDF3/OLIG3 | 18 |
| BP | GO:0035270 | endocrine system development | 12/363 | 127/18670 | 0.00 | 0.00 | 0.00 | ANXA1/HOXB3/HOXA5/PITX1/HOXA3/NKX2-5/OTP/PITX2/WT1/PDX1/FOXA2/HNF4A | 12 |
| CC | GO:0035580 | specific granule lumen | 8/372 | 62/19717 | 0.00 | 0.00 | 0.00 | CHI3L1/LTF/LYZ/PTX3/HP/RETN/CAMP/MMP8 | 8 |
| BP | GO:0030216 | keratinocyte differentiation | 19/363 | 305/18670 | 0.00 | 0.00 | 0.00 | ANXA1/PI3/CYP27B1/DSG2/VDR/HOXA7/WNT16/KRT75/KRT80/KRT14/KLK5/KRT6A/KRT31/SPRR1B/LIPN/SPRR3/KRT33B/KRT6C/SPRR2E | 19 |
| BP | GO:0090596 | sensory organ morphogenesis | 17/363 | 256/18670 | 0.00 | 0.00 | 0.00 | VEGFA/COL5A2/COL5A1/COL8A1/CELSR1/CTHRC1/MFAP2/CHRNA9/HOXA2/WNT16/HOXC13/HOXA1/TFAP2B/NKX3-2/PITX2/CLRN1/HMX3 | 17 |
| MF | GO:0005539 | glycosaminoglycan binding | 16/351 | 229/17697 | 0.00 | 0.00 | 0.00 | LTF/VEGFA/POSTN/THBS1/COL5A1/PCOLCE/SAA1/CXCL10/TNFAIP6/CCN4/MMP7/CXCL11/CXCL6/CCL7/EPYC/REG1B | 16 |
| BP | GO:0048608 | reproductive structure development | 23/363 | 431/18670 | 0.00 | 0.00 | 0.00 | VEGFA/ANXA1/PTX3/SDC1/LIF/GJB2/STC1/MMP19/E2F7/HOXA10/CYP27B1/HOXD13/VDR/HOXB13/PHLDA2/MAS1/HOXA9/TCF23/GATA4/WT1/RETN/SRY/CSF2 | 23 |
| MF | GO:0019838 | growth factor binding | 12/351 | 137/17697 | 0.00 | 0.00 | 0.00 | COL1A2/COL4A1/IGFBP2/COL1A1/COL3A1/THBS1/COL5A1/SRPX2/CCN4/ESM1/HTRA3/IL2RA | 12 |
| BP | GO:0061458 | reproductive system development | 23/363 | 434/18670 | 0.00 | 0.00 | 0.00 | VEGFA/ANXA1/PTX3/SDC1/LIF/GJB2/STC1/MMP19/E2F7/HOXA10/CYP27B1/HOXD13/VDR/HOXB13/PHLDA2/MAS1/HOXA9/TCF23/GATA4/WT1/RETN/SRY/CSF2 | 23 |
| BP | GO:0007369 | gastrulation | 14/363 | 185/18670 | 0.00 | 0.00 | 0.00 | COL4A2/COL5A2/COL5A1/COL8A1/MMP9/DKK1/HMGA2/FOXA2/ELF5/CER1/HNF4A/MMP8/TBX20/GDF3 | 14 |
| BP | GO:0060537 | muscle tissue development | 22/363 | 408/18670 | 0.00 | 0.00 | 0.00 | COL3A1/VEGFA/LOX/MEOX2/DMRTA2/SHOX2/CHRNA1/WNT10B/NOX4/DKK1/HOXD9/HOXD10/DSG2/PITX1/NKX2-5/TNNT2/ALPK2/GATA4/WT1/VGLL2/FGF3/TBX20 | 22 |
| MF | GO:0001158 | enhancer sequence-specific DNA binding | 11/351 | 119/17697 | 0.00 | 0.00 | 0.00 | MEOX2/HOXA5/HOXB7/HOXD13/HOXA7/HOXC6/TFAP2B/GATA4/HOXA6/HOXB5/HOXC5 | 11 |
| BP | GO:0032103 | positive regulation of response to external stimulus | 19/363 | 323/18670 | 0.00 | 0.00 | 0.00 | VEGFA/SERPINE1/THBS1/RARRES2/S100A9/CXCL8/PLA2G2A/CXCL10/CCN4/GBP5/CYP27B1/KLK7/TNFSF14/FPR2/IDO1/KLK5/CCL7/CREB3L3/MMP8 | 19 |
| BP | GO:0018149 | peptide cross-linking | 8/363 | 60/18670 | 0.00 | 0.00 | 0.00 | COL3A1/ANXA1/F13A1/THBS1/PI3/SPRR1B/SPRR3/SPRR2E | 8 |
| MF | GO:0008201 | heparin binding | 13/351 | 169/17697 | 0.00 | 0.00 | 0.00 | LTF/VEGFA/POSTN/THBS1/COL5A1/PCOLCE/SAA1/CXCL10/CCN4/MMP7/CXCL11/CXCL6/CCL7 | 13 |
| MF | GO:0045236 | CXCR chemokine receptor binding | 4/351 | 11/17697 | 0.00 | 0.00 | 0.00 | CXCL8/CXCL10/CXCL9/CXCL11 | 4 |
| MF | GO:0008009 | chemokine activity | 7/351 | 49/17697 | 0.00 | 0.00 | 0.00 | CXCL8/CXCL10/CXCL9/CXCL11/CCL20/CXCL6/CCL7 | 7 |
| BP | GO:0014706 | striated muscle tissue development | 21/363 | 390/18670 | 0.00 | 0.00 | 0.00 | VEGFA/LOX/MEOX2/DMRTA2/SHOX2/CHRNA1/WNT10B/NOX4/DKK1/HOXD9/HOXD10/DSG2/PITX1/NKX2-5/TNNT2/ALPK2/GATA4/WT1/VGLL2/FGF3/TBX20 | 21 |
| BP | GO:0044706 | multi-multicellular organism process | 15/363 | 222/18670 | 0.00 | 0.00 | 0.00 | IGFBP2/NAMPT/TIMP1/MMP9/LIF/GJB2/STC1/CYP27B1/MMP7/DSG2/VDR/IDO1/TCF23/EPYC/HMX3 | 15 |
| MF | GO:0005126 | cytokine receptor binding | 17/351 | 286/17697 | 0.00 | 0.00 | 0.00 | VEGFA/CXCL8/LIF/CXCL10/GDF10/GDF15/CLCF1/CXCL9/CXCL11/TNFSF14/CCL20/CXCL6/CD70/CCL7/IL36B/GDF3/CSF2 | 17 |
| CC | GO:0031983 | vesicle lumen | 18/372 | 339/19717 | 0.00 | 0.00 | 0.00 | CHI3L1/LTF/TIMP1/ANXA2/VEGFA/SERPINE1/F13A1/THBS1/FABP5/RARRES2/LYZ/S100A9/PTX3/SAA1/HP/RETN/CAMP/MMP8 | 18 |
| CC | GO:0060205 | cytoplasmic vesicle lumen | 18/372 | 338/19717 | 0.00 | 0.00 | 0.00 | CHI3L1/LTF/TIMP1/ANXA2/VEGFA/SERPINE1/F13A1/THBS1/FABP5/RARRES2/LYZ/S100A9/PTX3/SAA1/HP/RETN/CAMP/MMP8 | 18 |
| MF | GO:0035326 | enhancer binding | 11/351 | 133/17697 | 0.00 | 0.00 | 0.00 | MEOX2/HOXA5/HOXB7/HOXD13/HOXA7/HOXC6/TFAP2B/GATA4/HOXA6/HOXB5/HOXC5 | 11 |
| BP | GO:0030593 | neutrophil chemotaxis | 10/363 | 104/18670 | 0.00 | 0.00 | 0.00 | S100A9/CXCL8/SAA1/CXCL10/CXCL9/CXCL11/CCL20/CXCL6/CCL7/IL36B | 10 |
| BP | GO:0048665 | neuron fate specification | 6/363 | 34/18670 | 0.00 | 0.00 | 0.00 | DMRTA2/HOXC10/HOXD10/GSX2/LBX1/OLIG3 | 6 |
| BP | GO:0048732 | gland development | 22/363 | 434/18670 | 0.00 | 0.00 | 0.00 | VEGFA/ANXA1/HOXB3/E2F7/HOXA5/HOXD9/PITX1/HOXA3/HOXD13/NKX2-5/OTP/HAND2/VDR/HOXB13/HOXA9/PITX2/WT1/HOXB9/FOXB1/PDX1/ELF5/HNF4A | 22 |
| BP | GO:0002690 | positive regulation of leukocyte chemotaxis | 9/363 | 87/18670 | 0.00 | 0.00 | 0.00 | VEGFA/SERPINE1/THBS1/RARRES2/CXCL8/CXCL10/TNFSF14/FPR2/CCL7 | 9 |
| BP | GO:0001893 | maternal placenta development | 6/363 | 35/18670 | 0.00 | 0.00 | 0.00 | LIF/GJB2/STC1/CYP27B1/VDR/TCF23 | 6 |
| CC | GO:0034774 | secretory granule lumen | 17/372 | 321/19717 | 0.00 | 0.00 | 0.00 | CHI3L1/LTF/TIMP1/ANXA2/VEGFA/SERPINE1/F13A1/THBS1/FABP5/RARRES2/LYZ/S100A9/PTX3/HP/RETN/CAMP/MMP8 | 17 |
| BP | GO:0003417 | growth plate cartilage development | 6/363 | 36/18670 | 0.00 | 0.00 | 0.00 | ANXA2/COL6A2/COL6A3/STC1/MMP13/CER1 | 6 |
| BP | GO:0051146 | striated muscle cell differentiation | 17/363 | 293/18670 | 0.00 | 0.00 | 0.00 | VEGFA/LOX/ADAM12/SDC1/CXCL10/GDF15/SHOX2/WNT10B/CXCL9/NOX4/DKK1/NKX2-5/TNNT2/ALPK2/TNFSF14/GATA4/WT1 | 17 |
| BP | GO:0006959 | humoral immune response | 19/363 | 356/18670 | 0.00 | 0.00 | 0.00 | LTF/RARRES2/LYZ/S100A9/CXCL8/PLA2G2A/CXCL10/TREM1/FCGR2B/PI3/CXCL9/CXCL11/KLK7/CXCL6/KLK5/KRT6A/CAMP/REG1B/H2BS1 | 19 |
| BP | GO:0060350 | endochondral bone morphogenesis | 8/363 | 73/18670 | 0.00 | 0.00 | 0.00 | COL1A1/ANXA2/COL6A2/COL6A3/SHOX2/STC1/MMP13/CER1 | 8 |
| BP | GO:0001895 | retina homeostasis | 8/363 | 74/18670 | 0.00 | 0.01 | 0.00 | LTF/LYZ/CNGB1/POTEE/POTEF/POTEJ/POTEI/CLRN1 | 8 |
| BP | GO:1990266 | neutrophil migration | 10/363 | 118/18670 | 0.00 | 0.01 | 0.01 | S100A9/CXCL8/SAA1/CXCL10/CXCL9/CXCL11/CCL20/CXCL6/CCL7/IL36B | 10 |
| BP | GO:0010951 | negative regulation of endopeptidase activity | 15/363 | 250/18670 | 0.00 | 0.01 | 0.01 | LTF/TIMP1/VEGFA/SERPINE1/THBS1/COL6A3/MMP9/PI3/DPEP1/RARRES1/TFPI2/TNFSF14/TFAP2B/SERPINB2/MAGEA3 | 15 |
| BP | GO:0007605 | sensory perception of sound | 11/363 | 145/18670 | 0.00 | 0.01 | 0.01 | COL1A1/GABRB2/GJB2/EYA4/CHRNA9/PAX3/HOXA1/OTOR/CLRN1/EPYC/CEACAM16 | 11 |
| BP | GO:1900120 | regulation of receptor binding | 5/363 | 26/18670 | 0.00 | 0.01 | 0.01 | ANXA2/LOX/MMP9/HOXA3/PHLDA2 | 5 |
| BP | GO:0042119 | neutrophil activation | 23/363 | 498/18670 | 0.00 | 0.01 | 0.01 | CHI3L1/LTF/ANXA2/FABP5/PLAU/LYZ/S100A9/CXCL8/MMP9/PTX3/HP/CLEC5A/TNFAIP6/FCGR2B/FPR2/CLEC12A/CXCL6/MCEMP1/ADGRE3/RETN/CAMP/MMP8/CALML5 | 23 |
| BP | GO:0031424 | keratinization | 14/363 | 224/18670 | 0.00 | 0.01 | 0.01 | PI3/DSG2/KRT75/KRT80/KRT14/KLK5/KRT6A/KRT31/SPRR1B/LIPN/SPRR3/KRT33B/KRT6C/SPRR2E | 14 |
| BP | GO:0001704 | formation of primary germ layer | 10/363 | 121/18670 | 0.00 | 0.01 | 0.01 | COL4A2/COL5A2/COL5A1/COL8A1/MMP9/DKK1/HMGA2/ELF5/MMP8/TBX20 | 10 |
| BP | GO:0035136 | forelimb morphogenesis | 6/363 | 41/18670 | 0.00 | 0.01 | 0.01 | SHOX2/EN1/HOXD9/HOXD10/HOXA9/TFAP2B | 6 |
| BP | GO:0001708 | cell fate specification | 9/363 | 99/18670 | 0.00 | 0.01 | 0.01 | DMRTA2/HOXC10/DKK1/HOXD10/GSX2/LBX1/FOXA2/TBX20/OLIG3 | 9 |
| MF | GO:0002020 | protease binding | 10/351 | 128/17697 | 0.00 | 0.01 | 0.01 | COL1A2/TIMP1/COL1A1/ANXA2/COL3A1/SERPINE1/DPP4/BDKRB2/CD70/MAGEA3 | 10 |
| BP | GO:0007631 | feeding behavior | 9/363 | 101/18670 | 0.00 | 0.01 | 0.01 | GRIN1/CCK/GDF15/EN1/HAND2/CARTPT/GAL/DMBX1/RETN | 9 |
| BP | GO:0110110 | positive regulation of animal organ morphogenesis | 8/363 | 81/18670 | 0.00 | 0.01 | 0.01 | VEGFA/LIF/DKK1/HOXB7/HAND2/VDR/HOXC11/WT1 | 8 |
| BP | GO:0042730 | fibrinolysis | 5/363 | 28/18670 | 0.00 | 0.01 | 0.01 | ANXA2/SERPINE1/THBS1/PLAU/SERPINB2 | 5 |
| BP | GO:0003416 | endochondral bone growth | 6/363 | 44/18670 | 0.00 | 0.01 | 0.01 | ANXA2/COL6A2/COL6A3/STC1/MMP13/CER1 | 6 |
| BP | GO:0002687 | positive regulation of leukocyte migration | 10/363 | 128/18670 | 0.00 | 0.01 | 0.01 | VEGFA/SERPINE1/THBS1/RARRES2/CXCL8/CXCL10/TNFSF14/FPR2/CCL20/CCL7 | 10 |
| BP | GO:0010466 | negative regulation of peptidase activity | 15/363 | 262/18670 | 0.00 | 0.01 | 0.01 | LTF/TIMP1/VEGFA/SERPINE1/THBS1/COL6A3/MMP9/PI3/DPEP1/RARRES1/TFPI2/TNFSF14/TFAP2B/SERPINB2/MAGEA3 | 15 |
| MF | GO:0004252 | serine-type endopeptidase activity | 11/351 | 160/17697 | 0.00 | 0.01 | 0.01 | LTF/PLAU/MMP9/HP/DPP4/HTRA3/MMP7/KLK7/TMPRSS7/KLK5/MMP8 | 11 |
| MF | GO:0005518 | collagen binding | 7/351 | 67/17697 | 0.00 | 0.01 | 0.01 | TGFBI/THBS1/PCOLCE/MMP9/LUM/LRRC15/MMP13 | 7 |
| MF | GO:0042379 | chemokine receptor binding | 7/351 | 66/17697 | 0.00 | 0.01 | 0.01 | CXCL8/CXCL10/CXCL9/CXCL11/CCL20/CXCL6/CCL7 | 7 |
| BP | GO:0060536 | cartilage morphogenesis | 5/363 | 29/18670 | 0.00 | 0.01 | 0.01 | COL6A2/COL6A3/STC1/HOXA5/HAND2 | 5 |
| BP | GO:0048871 | multicellular organismal homeostasis | 22/363 | 485/18670 | 0.00 | 0.01 | 0.01 | LTF/VEGFA/FABP5/LYZ/TNFRSF11B/MFAP2/G0S2/HOXC10/TRPM8/WNT10B/NOX4/SCNN1B/CARTPT/CNGB1/TFAP2B/CIDEA/POTEE/POTEF/POTEJ/POTEI/CLRN1/RHAG | 22 |
| CC | GO:0005604 | basement membrane | 8/372 | 95/19717 | 0.00 | 0.01 | 0.01 | COL4A2/COL4A1/TIMP1/ANXA2/TGFBI/COL5A1/COL8A1/LOXL1 | 8 |
| MF | GO:0008083 | growth factor activity | 11/351 | 163/17697 | 0.00 | 0.01 | 0.01 | TIMP1/VEGFA/LIF/GDF10/GDF15/CLCF1/DKK1/FGF3/FGF19/GDF3/CSF2 | 11 |
| BP | GO:0001655 | urogenital system development | 17/363 | 330/18670 | 0.00 | 0.01 | 0.01 | COL4A1/VEGFA/ANXA1/MMP9/SDC1/LIF/CEP55/HOXB7/HOXD13/HOXB13/HOXD11/HOXC11/TFAP2B/WT1/FOXB1/CER1/SIM1 | 17 |
| BP | GO:0030574 | collagen catabolic process | 6/363 | 47/18670 | 0.00 | 0.01 | 0.01 | MMP9/MMP19/MMP11/MMP7/MMP13/MMP8 | 6 |
| BP | GO:0098868 | bone growth | 6/363 | 47/18670 | 0.00 | 0.01 | 0.01 | ANXA2/COL6A2/COL6A3/STC1/MMP13/CER1 | 6 |
| BP | GO:0001503 | ossification | 19/363 | 398/18670 | 0.00 | 0.01 | 0.01 | LTF/COL1A2/COL1A1/COL5A2/LOX/GDF10/CTHRC1/SHOX2/IBSP/CLEC5A/STC1/CCN4/WNT10B/CYP27B1/DKK1/HAND2/HOXA2/MMP13/CER1 | 19 |
| BP | GO:0061371 | determination of heart left/right asymmetry | 7/363 | 67/18670 | 0.00 | 0.01 | 0.01 | NKX2-5/DNAH11/HAND2/GATA4/LBX1/CER1/TBX20 | 7 |
| BP | GO:0050900 | leukocyte migration | 22/363 | 499/18670 | 0.00 | 0.01 | 0.01 | COL1A2/COL1A1/VEGFA/ANXA1/SERPINE1/THBS1/RARRES2/S100A9/CXCL8/SDC1/SAA1/CXCL10/TREM1/CXCL9/CXCL11/HOXA7/TNFSF14/FPR2/CCL20/CXCL6/CCL7/IL36B | 22 |
| BP | GO:0060348 | bone development | 13/363 | 217/18670 | 0.00 | 0.01 | 0.01 | LTF/COL1A1/ANXA2/COL6A2/COL6A3/LOX/SHOX2/STC1/CCN4/HOXB4/PITX2/MMP13/CER1 | 13 |
| BP | GO:0031214 | biomineral tissue development | 11/363 | 163/18670 | 0.00 | 0.01 | 0.01 | LTF/COL1A2/COL1A1/ANXA2/LOX/IBSP/WNT10B/CYP27B1/MMP13/WDR72/CER1 | 11 |
| BP | GO:0071674 | mononuclear cell migration | 8/363 | 90/18670 | 0.00 | 0.01 | 0.01 | ANXA1/SERPINE1/THBS1/RARRES2/CXCL10/FPR2/CCL20/CCL7 | 8 |
| BP | GO:0009953 | dorsal/ventral pattern formation | 8/363 | 90/18670 | 0.00 | 0.01 | 0.01 | HOXB2/EN1/SP8/GSX2/HOXA2/HOXD11/CER1/TBX20 | 8 |
| BP | GO:0060485 | mesenchyme development | 15/363 | 278/18670 | 0.00 | 0.02 | 0.01 | COL1A1/PDPN/S100A4/HOXA5/NKX2-5/HAND2/HMGA2/WNT16/PITX2/GATA4/WT1/FOXA2/CER1/TBX20/FGF19 | 15 |
| BP | GO:0002688 | regulation of leukocyte chemotaxis | 9/363 | 114/18670 | 0.00 | 0.02 | 0.01 | VEGFA/SERPINE1/THBS1/RARRES2/CXCL8/CXCL10/TNFSF14/FPR2/CCL7 | 9 |
| BP | GO:0060349 | bone morphogenesis | 9/363 | 114/18670 | 0.00 | 0.02 | 0.01 | LTF/COL1A1/ANXA2/COL6A2/COL6A3/SHOX2/STC1/MMP13/CER1 | 9 |
| BP | GO:0050954 | sensory perception of mechanical stimulus | 11/363 | 165/18670 | 0.00 | 0.02 | 0.01 | COL1A1/GABRB2/GJB2/EYA4/CHRNA9/PAX3/HOXA1/OTOR/CLRN1/EPYC/CEACAM16 | 11 |
| BP | GO:0048536 | spleen development | 5/363 | 33/18670 | 0.00 | 0.02 | 0.01 | NKX2-5/HOXB4/NKX3-2/PITX2/BARX1 | 5 |
| BP | GO:0032963 | collagen metabolic process | 9/363 | 115/18670 | 0.00 | 0.02 | 0.01 | COL1A2/COL1A1/COL5A1/MMP9/MMP19/MMP11/MMP7/MMP13/MMP8 | 9 |
| BP | GO:2000117 | negative regulation of cysteine-type endopeptidase activity | 8/363 | 92/18670 | 0.00 | 0.02 | 0.01 | LTF/VEGFA/THBS1/MMP9/DPEP1/TNFSF14/TFAP2B/MAGEA3 | 8 |
| BP | GO:0033273 | response to vitamin | 8/363 | 93/18670 | 0.00 | 0.02 | 0.01 | COL1A1/POSTN/CXCL10/STC1/CYP27B1/VDR/GATA4/PDX1 | 8 |
| BP | GO:0021522 | spinal cord motor neuron differentiation | 5/363 | 34/18670 | 0.00 | 0.02 | 0.01 | HOXC10/HOXD10/LBX1/TBX20/OLIG3 | 5 |
| BP | GO:0002685 | regulation of leukocyte migration | 12/363 | 196/18670 | 0.00 | 0.02 | 0.01 | VEGFA/ANXA1/SERPINE1/THBS1/RARRES2/CXCL8/CXCL10/HOXA7/TNFSF14/FPR2/CCL20/CCL7 | 12 |
| BP | GO:0001763 | morphogenesis of a branching structure | 12/363 | 196/18670 | 0.00 | 0.02 | 0.01 | COL4A1/VEGFA/CELSR1/SHOX2/HOXA5/HOXB7/HOXD13/VDR/HOXB13/HOXD11/WT1/TBX20 | 12 |
| BP | GO:0060538 | skeletal muscle organ development | 11/363 | 169/18670 | 0.00 | 0.02 | 0.01 | MEOX2/DMRTA2/SHOX2/CHRNA1/WNT10B/DKK1/HOXD9/HOXD10/PITX1/WT1/VGLL2 | 11 |
| BP | GO:0071559 | response to transforming growth factor beta | 14/363 | 255/18670 | 0.00 | 0.02 | 0.01 | COL4A2/COL1A2/COL1A1/COL3A1/POSTN/FMOD/THBS1/LOX/MXRA5/GDF10/GDF15/HTRA3/NOX4/CIDEA | 14 |
| BP | GO:0007178 | transmembrane receptor protein serine/threonine kinase signaling pathway | 17/363 | 349/18670 | 0.00 | 0.02 | 0.02 | COL1A2/COL3A1/FMOD/THBS1/LOX/GDF10/GDF15/HTRA3/DKK1/NKX2-5/TFAP2B/GATA4/CIDEA/CER1/HNF4A/TBX20/GDF3 | 17 |
| MF | GO:0000980 | RNA polymerase II distal enhancer sequence-specific DNA binding | 8/351 | 99/17697 | 0.00 | 0.02 | 0.02 | MEOX2/HOXA5/HOXB7/HOXA7/HOXC6/HOXA6/HOXB5/HOXC5 | 8 |
| MF | GO:0004866 | endopeptidase inhibitor activity | 11/351 | 175/17697 | 0.00 | 0.02 | 0.02 | LTF/TIMP1/SERPINE1/COL6A3/PI3/DPEP1/RARRES1/TFPI2/TNFSF14/TFAP2B/SERPINB2 | 11 |
| BP | GO:0007368 | determination of left/right symmetry | 9/363 | 119/18670 | 0.00 | 0.02 | 0.02 | NKX2-5/DNAH11/HAND2/NKX3-2/PITX2/GATA4/LBX1/CER1/TBX20 | 9 |
| BP | GO:0003215 | cardiac right ventricle morphogenesis | 4/363 | 20/18670 | 0.00 | 0.02 | 0.02 | NKX2-5/HAND2/GATA4/TBX20 | 4 |
| BP | GO:0042692 | muscle cell differentiation | 18/363 | 385/18670 | 0.00 | 0.02 | 0.02 | VEGFA/LOX/ADAM12/SDC1/CXCL10/GDF15/SHOX2/WNT10B/CXCL9/NOX4/DKK1/NKX2-5/TNNT2/ALPK2/TNFSF14/GATA4/WT1/FBXO40 | 18 |
| BP | GO:0003007 | heart morphogenesis | 14/363 | 259/18670 | 0.00 | 0.02 | 0.02 | VEGFA/COL5A1/SHOX2/DKK1/NKX2-5/DNAH11/TNNT2/HAND2/WNT16/ALPK2/PITX2/GATA4/LBX1/TBX20 | 14 |
| BP | GO:0055123 | digestive system development | 10/363 | 146/18670 | 0.00 | 0.02 | 0.02 | COL3A1/RARRES2/CXCL8/SHOX2/HOXA5/HOXD13/NKX3-2/GATA4/BARX1/PDX1 | 10 |
| BP | GO:1990868 | response to chemokine | 8/363 | 97/18670 | 0.00 | 0.02 | 0.02 | CXCL8/LOX/CXCL10/CXCL9/CXCL11/CCL20/CXCL6/CCL7 | 8 |
| BP | GO:0051591 | response to cAMP | 8/363 | 97/18670 | 0.00 | 0.02 | 0.02 | COL1A1/SDC1/STC1/MMP19/WNT10B/NOX4/WT1/AGXT | 8 |
| BP | GO:1990869 | cellular response to chemokine | 8/363 | 97/18670 | 0.00 | 0.02 | 0.02 | CXCL8/LOX/CXCL10/CXCL9/CXCL11/CCL20/CXCL6/CCL7 | 8 |
| BP | GO:0021515 | cell differentiation in spinal cord | 6/363 | 54/18670 | 0.00 | 0.02 | 0.02 | HOXC10/HOXD10/GSX2/LBX1/TBX20/OLIG3 | 6 |
| MF | GO:0050840 | extracellular matrix binding | 6/351 | 57/17697 | 0.00 | 0.02 | 0.02 | ANXA2/VEGFA/TGFBI/THBS1/CD248/LRRC15 | 6 |
| MF | GO:0008236 | serine-type peptidase activity | 11/351 | 182/17697 | 0.00 | 0.02 | 0.02 | LTF/PLAU/MMP9/HP/DPP4/HTRA3/MMP7/KLK7/TMPRSS7/KLK5/MMP8 | 11 |
| MF | GO:0030414 | peptidase inhibitor activity | 11/351 | 182/17697 | 0.00 | 0.02 | 0.02 | LTF/TIMP1/SERPINE1/COL6A3/PI3/DPEP1/RARRES1/TFPI2/TNFSF14/TFAP2B/SERPINB2 | 11 |
| MF | GO:0061135 | endopeptidase regulator activity | 11/351 | 182/17697 | 0.00 | 0.02 | 0.02 | LTF/TIMP1/SERPINE1/COL6A3/PI3/DPEP1/RARRES1/TFPI2/TNFSF14/TFAP2B/SERPINB2 | 11 |
| BP | GO:0061041 | regulation of wound healing | 10/363 | 148/18670 | 0.00 | 0.02 | 0.02 | ANXA2/ANXA1/PDPN/SERPINE1/THBS1/TNFRSF12A/PLAU/CCN4/SERPINB2/FOXA2 | 10 |
| BP | GO:1901739 | regulation of myoblast fusion | 4/363 | 21/18670 | 0.00 | 0.02 | 0.02 | CXCL10/GDF15/CXCL9/TNFSF14 | 4 |
| BP | GO:0002062 | chondrocyte differentiation | 9/363 | 123/18670 | 0.00 | 0.02 | 0.02 | ANXA2/COL6A2/TGFBI/COL6A3/SHOX2/CCN4/WNT10B/HMGA2/NKX3-2 | 9 |
| BP | GO:0071542 | dopaminergic neuron differentiation | 5/363 | 37/18670 | 0.00 | 0.02 | 0.02 | VEGFA/DMRTA2/EN1/DKK1/FOXA2 | 5 |
| MF | GO:0022824 | transmitter-gated ion channel activity | 6/351 | 61/17697 | 0.00 | 0.02 | 0.02 | GRIN1/GABRB2/CHRNA1/CHRNA9/HTR3B/GABRA6 | 6 |
| MF | GO:0022835 | transmitter-gated channel activity | 6/351 | 61/17697 | 0.00 | 0.02 | 0.02 | GRIN1/GABRB2/CHRNA1/CHRNA9/HTR3B/GABRA6 | 6 |
| MF | GO:0017171 | serine hydrolase activity | 11/351 | 186/17697 | 0.00 | 0.02 | 0.02 | LTF/PLAU/MMP9/HP/DPP4/HTRA3/MMP7/KLK7/TMPRSS7/KLK5/MMP8 | 11 |
| MF | GO:1901681 | sulfur compound binding | 13/351 | 250/17697 | 0.00 | 0.02 | 0.02 | LTF/VEGFA/POSTN/THBS1/COL5A1/PCOLCE/SAA1/CXCL10/CCN4/MMP7/CXCL11/CXCL6/CCL7 | 13 |
| MF | GO:0061134 | peptidase regulator activity | 12/351 | 219/17697 | 0.00 | 0.02 | 0.02 | LTF/TIMP1/SERPINE1/PCOLCE/COL6A3/PI3/DPEP1/RARRES1/TFPI2/TNFSF14/TFAP2B/SERPINB2 | 12 |
| MF | GO:0001664 | G protein-coupled receptor binding | 14/351 | 280/17697 | 0.00 | 0.02 | 0.02 | CXCL8/SAA1/CXCL10/CTHRC1/BDKRB2/WNT10B/CXCL9/MARCO/CXCL11/WNT16/GAL/CCL20/CXCL6/CCL7 | 14 |
| MF | GO:0033613 | activating transcription factor binding | 7/351 | 85/17697 | 0.00 | 0.02 | 0.02 | HOXB4/HAND2/HOXA4/PITX2/GATA4/HNF4A/TBX20 | 7 |
| BP | GO:0008344 | adult locomotory behavior | 7/363 | 77/18670 | 0.00 | 0.02 | 0.02 | GRIN1/EN1/HOXD9/HOXD10/HOXB8/DMBX1/FOXA2 | 7 |
| BP | GO:0048771 | tissue remodeling | 11/363 | 179/18670 | 0.00 | 0.02 | 0.02 | TIMP1/ANXA1/LIF/CTHRC1/TNFRSF11B/NOX4/HOXA3/HAND2/CARTPT/VDR/WNT16 | 11 |
| BP | GO:0030878 | thyroid gland development | 4/363 | 22/18670 | 0.00 | 0.02 | 0.02 | HOXB3/HOXA5/HOXA3/NKX2-5 | 4 |
| BP | GO:1900122 | positive regulation of receptor binding | 3/363 | 10/18670 | 0.00 | 0.02 | 0.02 | ANXA2/MMP9/HOXA3 | 3 |
| BP | GO:0042742 | defense response to bacterium | 16/363 | 330/18670 | 0.00 | 0.02 | 0.02 | LTF/SERPINE1/RARRES2/LYZ/S100A9/PLA2G2A/HP/KLK7/FPR2/CCL20/CXCL6/DEFB119/KLK5/KRT6A/CAMP/H2BS1 | 16 |
| BP | GO:0038063 | collagen-activated tyrosine kinase receptor signaling pathway | 3/363 | 10/18670 | 0.00 | 0.02 | 0.02 | COL4A2/COL4A1/COL1A1 | 3 |
| BP | GO:0060923 | cardiac muscle cell fate commitment | 3/363 | 10/18670 | 0.00 | 0.02 | 0.02 | DKK1/NKX2-5/WT1 | 3 |
| BP | GO:0031667 | response to nutrient levels | 21/363 | 499/18670 | 0.00 | 0.02 | 0.02 | IGFBP2/NAMPT/COL1A1/GRIN1/POSTN/CCK/PRKCG/CXCL10/GDF15/STC1/TNFRSF11B/CYP27B1/MMP7/CARTPT/VDR/MAP1LC3C/GATA4/PDX1/FOXA2/CLPSL1/GDF3 | 21 |
| BP | GO:0002446 | neutrophil mediated immunity | 21/363 | 499/18670 | 0.00 | 0.02 | 0.02 | CHI3L1/LTF/ANXA2/FABP5/PLAU/LYZ/S100A9/MMP9/PTX3/HP/CLEC5A/TNFAIP6/FPR2/CLEC12A/CXCL6/MCEMP1/ADGRE3/RETN/CAMP/MMP8/CALML5 | 21 |
| BP | GO:0009612 | response to mechanical stimulus | 12/363 | 210/18670 | 0.00 | 0.02 | 0.02 | CHI3L1/IGFBP2/COL1A1/COL3A1/POSTN/THBS1/CXCL10/CHRNA9/MMP7/TNFSF14/GATA4/RETN | 12 |
| BP | GO:0030193 | regulation of blood coagulation | 7/363 | 79/18670 | 0.00 | 0.02 | 0.02 | ANXA2/PDPN/SERPINE1/THBS1/PLAU/SERPINB2/FOXA2 | 7 |
| BP | GO:0061138 | morphogenesis of a branching epithelium | 11/363 | 182/18670 | 0.00 | 0.02 | 0.02 | COL4A1/VEGFA/CELSR1/HOXA5/HOXB7/HOXD13/VDR/HOXB13/HOXD11/WT1/TBX20 | 11 |
| BP | GO:0009855 | determination of bilateral symmetry | 9/363 | 128/18670 | 0.00 | 0.02 | 0.02 | NKX2-5/DNAH11/HAND2/NKX3-2/PITX2/GATA4/LBX1/CER1/TBX20 | 9 |
| BP | GO:1900046 | regulation of hemostasis | 7/363 | 80/18670 | 0.00 | 0.03 | 0.02 | ANXA2/PDPN/SERPINE1/THBS1/PLAU/SERPINB2/FOXA2 | 7 |
| BP | GO:0009799 | specification of symmetry | 9/363 | 129/18670 | 0.00 | 0.03 | 0.02 | NKX2-5/DNAH11/HAND2/NKX3-2/PITX2/GATA4/LBX1/CER1/TBX20 | 9 |
| BP | GO:0007596 | blood coagulation | 16/363 | 336/18670 | 0.00 | 0.03 | 0.02 | COL1A2/COL1A1/ANXA2/COL3A1/PDPN/SERPINE1/F13A1/THBS1/PLAU/PRKCG/SAA1/TFPI2/GATA4/SERPINB2/FOXA2/HNF4A | 16 |
| BP | GO:0002517 | T cell tolerance induction | 3/363 | 11/18670 | 0.00 | 0.03 | 0.02 | IL2RA/IDO1/ICOS | 3 |
| BP | GO:1905941 | positive regulation of gonad development | 3/363 | 11/18670 | 0.00 | 0.03 | 0.02 | WT1/RETN/SRY | 3 |
| BP | GO:0021510 | spinal cord development | 8/363 | 106/18670 | 0.00 | 0.03 | 0.02 | HOXC10/HOXD10/GSX2/HOXB8/LBX1/FOXB1/TBX20/OLIG3 | 8 |
| BP | GO:0007599 | hemostasis | 16/363 | 341/18670 | 0.00 | 0.03 | 0.02 | COL1A2/COL1A1/ANXA2/COL3A1/PDPN/SERPINE1/F13A1/THBS1/PLAU/PRKCG/SAA1/TFPI2/GATA4/SERPINB2/FOXA2/HNF4A | 16 |
| BP | GO:0042476 | odontogenesis | 9/363 | 132/18670 | 0.00 | 0.03 | 0.03 | COL1A2/COL1A1/SERPINE1/SDC1/TNFRSF11B/HAND2/PITX2/KLK5/WDR72 | 9 |
| BP | GO:0007520 | myoblast fusion | 5/363 | 41/18670 | 0.00 | 0.03 | 0.03 | ADAM12/CXCL10/GDF15/CXCL9/TNFSF14 | 5 |
| BP | GO:0050817 | coagulation | 16/363 | 342/18670 | 0.00 | 0.03 | 0.03 | COL1A2/COL1A1/ANXA2/COL3A1/PDPN/SERPINE1/F13A1/THBS1/PLAU/PRKCG/SAA1/TFPI2/GATA4/SERPINB2/FOXA2/HNF4A | 16 |
| BP | GO:0048645 | animal organ formation | 6/363 | 61/18670 | 0.00 | 0.03 | 0.03 | DKK1/HOXA3/HAND2/HOXC11/NKX3-2/WT1 | 6 |
| BP | GO:0007519 | skeletal muscle tissue development | 10/363 | 160/18670 | 0.00 | 0.03 | 0.03 | MEOX2/DMRTA2/SHOX2/CHRNA1/WNT10B/DKK1/HOXD9/HOXD10/PITX1/VGLL2 | 10 |
| BP | GO:0007584 | response to nutrient | 12/363 | 219/18670 | 0.00 | 0.03 | 0.03 | IGFBP2/COL1A1/GRIN1/POSTN/CXCL10/STC1/TNFRSF11B/CYP27B1/VDR/GATA4/PDX1/FOXA2 | 12 |
| BP | GO:0032107 | regulation of response to nutrient levels | 4/363 | 25/18670 | 0.00 | 0.03 | 0.03 | CCK/PRKCG/CYP27B1/CARTPT | 4 |
| BP | GO:0032104 | regulation of response to extracellular stimulus | 4/363 | 25/18670 | 0.00 | 0.03 | 0.03 | CCK/PRKCG/CYP27B1/CARTPT | 4 |
| BP | GO:0010830 | regulation of myotube differentiation | 6/363 | 62/18670 | 0.00 | 0.03 | 0.03 | CXCL10/GDF15/SHOX2/CXCL9/NKX2-5/TNFSF14 | 6 |
| BP | GO:0010470 | regulation of gastrulation | 5/363 | 42/18670 | 0.00 | 0.03 | 0.03 | COL5A2/COL5A1/DKK1/FOXA2/HNF4A | 5 |
| BP | GO:0050818 | regulation of coagulation | 7/363 | 84/18670 | 0.00 | 0.03 | 0.03 | ANXA2/PDPN/SERPINE1/THBS1/PLAU/SERPINB2/FOXA2 | 7 |
| BP | GO:0031639 | plasminogen activation | 4/363 | 25/18670 | 0.00 | 0.03 | 0.03 | ANXA2/SERPINE1/THBS1/PLAU | 4 |
| BP | GO:0043154 | negative regulation of cysteine-type endopeptidase activity involved in apoptotic process | 7/363 | 84/18670 | 0.00 | 0.03 | 0.03 | VEGFA/THBS1/MMP9/DPEP1/TNFSF14/TFAP2B/MAGEA3 | 7 |
| BP | GO:0045445 | myoblast differentiation | 7/363 | 84/18670 | 0.00 | 0.03 | 0.03 | SDC1/CXCL10/WNT10B/CXCL9/PITX1/TNFSF14/GDF3 | 7 |
| BP | GO:0048565 | digestive tract development | 9/363 | 134/18670 | 0.00 | 0.03 | 0.03 | COL3A1/RARRES2/CXCL8/SHOX2/HOXA5/HOXD13/NKX3-2/GATA4/PDX1 | 9 |
| BP | GO:0071560 | cellular response to transforming growth factor beta stimulus | 13/363 | 249/18670 | 0.00 | 0.03 | 0.03 | COL4A2/COL1A2/COL1A1/COL3A1/POSTN/FMOD/THBS1/LOX/GDF10/GDF15/HTRA3/NOX4/CIDEA | 13 |
| BP | GO:0050921 | positive regulation of chemotaxis | 9/363 | 135/18670 | 0.00 | 0.03 | 0.03 | VEGFA/SERPINE1/THBS1/RARRES2/CXCL8/CXCL10/TNFSF14/FPR2/CCL7 | 9 |
| MF | GO:0005520 | insulin-like growth factor binding | 4/351 | 28/17697 | 0.00 | 0.03 | 0.03 | IGFBP2/CCN4/ESM1/HTRA3 | 4 |
| CC | GO:0001533 | cornified envelope | 6/372 | 65/19717 | 0.00 | 0.03 | 0.03 | ANXA1/PI3/DSG2/SPRR1B/SPRR3/SPRR2E | 6 |
| MF | GO:1904315 | transmitter-gated ion channel activity involved in regulation of postsynaptic membrane potential | 5/351 | 47/17697 | 0.00 | 0.03 | 0.03 | GRIN1/GABRB2/CHRNA1/CHRNA9/GABRA6 | 5 |
| MF | GO:0030594 | neurotransmitter receptor activity | 8/351 | 117/17697 | 0.00 | 0.03 | 0.03 | GRIN1/GABRB2/CHRNA1/CHRNA9/HTR5A/HTR1E/HTR3B/GABRA6 | 8 |
| BP | GO:0043312 | neutrophil degranulation | 20/363 | 485/18670 | 0.00 | 0.03 | 0.03 | CHI3L1/LTF/ANXA2/FABP5/PLAU/LYZ/S100A9/MMP9/PTX3/HP/CLEC5A/TNFAIP6/FPR2/CLEC12A/MCEMP1/ADGRE3/RETN/CAMP/MMP8/CALML5 | 20 |
| BP | GO:0048934 | peripheral nervous system neuron differentiation | 3/363 | 12/18670 | 0.00 | 0.03 | 0.03 | HOXD9/HOXD10/HAND2 | 3 |
| BP | GO:0048935 | peripheral nervous system neuron development | 3/363 | 12/18670 | 0.00 | 0.03 | 0.03 | HOXD9/HOXD10/HAND2 | 3 |
| BP | GO:0003207 | cardiac chamber formation | 3/363 | 12/18670 | 0.00 | 0.03 | 0.03 | NKX2-5/HAND2/TBX20 | 3 |
| BP | GO:0035821 | modification of morphology or physiology of other organism | 10/363 | 164/18670 | 0.00 | 0.03 | 0.03 | LTF/ANXA2/LYZ/S100A9/PTX3/HMGA2/CXCL6/KRT6A/CAMP/REG1B | 10 |
| BP | GO:0052547 | regulation of peptidase activity | 19/363 | 452/18670 | 0.00 | 0.03 | 0.03 | LTF/TIMP1/VEGFA/SERPINE1/GRIN1/THBS1/PCOLCE/COL6A3/S100A9/CCK/MMP9/PI3/DPEP1/RARRES1/TFPI2/TNFSF14/TFAP2B/SERPINB2/MAGEA3 | 19 |
| BP | GO:0002283 | neutrophil activation involved in immune response | 20/363 | 488/18670 | 0.00 | 0.03 | 0.03 | CHI3L1/LTF/ANXA2/FABP5/PLAU/LYZ/S100A9/MMP9/PTX3/HP/CLEC5A/TNFAIP6/FPR2/CLEC12A/MCEMP1/ADGRE3/RETN/CAMP/MMP8/CALML5 | 20 |
| BP | GO:0048247 | lymphocyte chemotaxis | 6/363 | 64/18670 | 0.00 | 0.03 | 0.03 | SAA1/CXCL10/CXCL11/TNFSF14/CCL20/CCL7 | 6 |
| CC | GO:0005667 | transcription factor complex | 16/372 | 365/19717 | 0.00 | 0.03 | 0.03 | E2F7/HOXA10/PITX1/NKX2-5/HAND2/VDR/HOXB13/HOXA9/SIX6/PITX2/GATA4/DMBX1/HOXB9/LBX1/SRY/HOXD12 | 16 |
| BP | GO:0014902 | myotube differentiation | 8/363 | 112/18670 | 0.00 | 0.03 | 0.03 | ADAM12/CXCL10/GDF15/SHOX2/WNT10B/CXCL9/NKX2-5/TNFSF14 | 8 |
| BP | GO:0001649 | osteoblast differentiation | 12/363 | 225/18670 | 0.00 | 0.03 | 0.03 | LTF/COL1A1/LOX/GDF10/CTHRC1/SHOX2/IBSP/CLEC5A/CCN4/WNT10B/HAND2/HOXA2 | 12 |
| BP | GO:0048663 | neuron fate commitment | 6/363 | 65/18670 | 0.00 | 0.03 | 0.03 | DMRTA2/HOXC10/HOXD10/GSX2/LBX1/OLIG3 | 6 |
| BP | GO:0002548 | monocyte chemotaxis | 6/363 | 65/18670 | 0.00 | 0.03 | 0.03 | ANXA1/SERPINE1/CXCL10/FPR2/CCL20/CCL7 | 6 |
| BP | GO:0070098 | chemokine-mediated signaling pathway | 7/363 | 88/18670 | 0.00 | 0.03 | 0.03 | CXCL8/CXCL10/CXCL9/CXCL11/CCL20/CXCL6/CCL7 | 7 |
| BP | GO:0035051 | cardiocyte differentiation | 10/363 | 167/18670 | 0.00 | 0.03 | 0.03 | ANXA2/VEGFA/NOX4/DKK1/NKX2-5/HAND2/ALPK2/PITX2/GATA4/WT1 | 10 |
| BP | GO:0007568 | aging | 15/363 | 321/18670 | 0.00 | 0.03 | 0.03 | COL4A2/IGFBP2/NAMPT/TIMP1/SERPINE1/GJB2/NOX4/DKK1/MMP7/HMGA2/WNT16/KRT14/PDX1/RETN/KRT33B | 15 |
| BP | GO:0001894 | tissue homeostasis | 12/363 | 227/18670 | 0.00 | 0.04 | 0.03 | LTF/VEGFA/LYZ/TNFRSF11B/NOX4/CARTPT/CNGB1/POTEE/POTEF/POTEJ/POTEI/CLRN1 | 12 |
| BP | GO:0072073 | kidney epithelium development | 9/363 | 140/18670 | 0.00 | 0.04 | 0.03 | VEGFA/SDC1/LIF/HOXB7/HOXD11/TFAP2B/WT1/CER1/SIM1 | 9 |
| BP | GO:0060562 | epithelial tube morphogenesis | 15/363 | 322/18670 | 0.00 | 0.04 | 0.03 | COL4A1/VEGFA/CXCL10/CELSR1/CTHRC1/HOXA5/HOXB7/NKX2-5/HAND2/VDR/HOXD11/GATA4/WT1/LBX1/TBX20 | 15 |
| BP | GO:0030282 | bone mineralization | 8/363 | 114/18670 | 0.00 | 0.04 | 0.03 | LTF/COL1A2/LOX/IBSP/WNT10B/CYP27B1/MMP13/CER1 | 8 |
| BP | GO:0052548 | regulation of endopeptidase activity | 18/363 | 425/18670 | 0.00 | 0.04 | 0.03 | LTF/TIMP1/VEGFA/SERPINE1/GRIN1/THBS1/COL6A3/S100A9/CCK/MMP9/PI3/DPEP1/RARRES1/TFPI2/TNFSF14/TFAP2B/SERPINB2/MAGEA3 | 18 |
| BP | GO:0021520 | spinal cord motor neuron cell fate specification | 3/363 | 13/18670 | 0.00 | 0.04 | 0.03 | HOXC10/HOXD10/OLIG3 | 3 |
| BP | GO:0070208 | protein heterotrimerization | 3/363 | 13/18670 | 0.00 | 0.04 | 0.03 | COL1A2/COL1A1/COL6A2 | 3 |
| BP | GO:0038065 | collagen-activated signaling pathway | 3/363 | 13/18670 | 0.00 | 0.04 | 0.03 | COL4A2/COL4A1/COL1A1 | 3 |
| BP | GO:0071675 | regulation of mononuclear cell migration | 5/363 | 46/18670 | 0.00 | 0.04 | 0.03 | SERPINE1/THBS1/RARRES2/CXCL10/FPR2 | 5 |
| BP | GO:0019731 | antibacterial humoral response | 5/363 | 46/18670 | 0.00 | 0.04 | 0.03 | LTF/KLK7/KLK5/CAMP/H2BS1 | 5 |
| MF | GO:0099529 | neurotransmitter receptor activity involved in regulation of postsynaptic membrane potential | 5/351 | 50/17697 | 0.00 | 0.04 | 0.03 | GRIN1/GABRB2/CHRNA1/CHRNA9/GABRA6 | 5 |
| BP | GO:0050918 | positive chemotaxis | 6/363 | 68/18670 | 0.00 | 0.04 | 0.03 | VEGFA/CXCL8/SAA1/CXCL10/SAA2/FPR2 | 6 |
| BP | GO:0051346 | negative regulation of hydrolase activity | 19/363 | 466/18670 | 0.00 | 0.04 | 0.03 | LTF/TIMP1/VEGFA/ANXA1/SERPINE1/SPOCD1/THBS1/COL6A3/MMP9/PTX3/PI3/DPEP1/RARRES1/TNNT2/TFPI2/TNFSF14/TFAP2B/SERPINB2/MAGEA3 | 19 |
| BP | GO:0010812 | negative regulation of cell-substrate adhesion | 6/363 | 68/18670 | 0.00 | 0.04 | 0.03 | COL1A1/SERPINE1/POSTN/GBP1/THBS1/HOXA7 | 6 |
| BP | GO:0045861 | negative regulation of proteolysis | 16/363 | 363/18670 | 0.00 | 0.04 | 0.04 | LTF/TIMP1/VEGFA/SERPINE1/THBS1/COL6A3/PRKCG/MMP9/PI3/DPEP1/RARRES1/TFPI2/TNFSF14/TFAP2B/SERPINB2/MAGEA3 | 16 |
| BP | GO:0042471 | ear morphogenesis | 8/363 | 118/18670 | 0.00 | 0.04 | 0.04 | CELSR1/CTHRC1/CHRNA9/HOXA2/HOXA1/NKX3-2/CLRN1/HMX3 | 8 |
| BP | GO:0060142 | regulation of syncytium formation by plasma membrane fusion | 4/363 | 29/18670 | 0.00 | 0.04 | 0.04 | CXCL10/GDF15/CXCL9/TNFSF14 | 4 |
| BP | GO:0010755 | regulation of plasminogen activation | 3/363 | 14/18670 | 0.00 | 0.04 | 0.04 | ANXA2/SERPINE1/THBS1 | 3 |
| BP | GO:0021602 | cranial nerve morphogenesis | 4/363 | 29/18670 | 0.00 | 0.04 | 0.04 | HOXB3/HOXB2/HOXA3/HOXA1 | 4 |
| BP | GO:0060911 | cardiac cell fate commitment | 3/363 | 14/18670 | 0.00 | 0.04 | 0.04 | DKK1/NKX2-5/WT1 | 3 |
| BP | GO:0021517 | ventral spinal cord development | 5/363 | 48/18670 | 0.00 | 0.04 | 0.04 | HOXC10/HOXD10/LBX1/TBX20/OLIG3 | 5 |
| MF | GO:0098960 | postsynaptic neurotransmitter receptor activity | 5/351 | 52/17697 | 0.00 | 0.05 | 0.04 | GRIN1/GABRB2/CHRNA1/CHRNA9/GABRA6 | 5 |
| MF | GO:0005230 | extracellular ligand-gated ion channel activity | 6/351 | 75/17697 | 0.00 | 0.05 | 0.04 | GRIN1/GABRB2/CHRNA1/CHRNA9/HTR3B/GABRA6 | 6 |
| BP | GO:0007548 | sex differentiation | 13/363 | 270/18670 | 0.00 | 0.05 | 0.04 | VEGFA/PTX3/SDC1/MMP19/HOXA10/HOXD13/MAS1/HOXA9/GATA4/WT1/RETN/HNF4A/SRY | 13 |
| BP | GO:0061326 | renal tubule development | 7/363 | 95/18670 | 0.00 | 0.05 | 0.04 | COL4A1/VEGFA/LIF/HOXB7/HOXD11/TFAP2B/WT1 | 7 |
| BP | GO:0003209 | cardiac atrium morphogenesis | 4/363 | 30/18670 | 0.00 | 0.05 | 0.04 | SHOX2/NKX2-5/GATA4/TBX20 | 4 |
