# Supplementary material for: High LYRM4-AS1 predicts poor prognosis in patients with glioma and correlates with immune infiltration
Source: PeerJ. 2023 Oct 3;11:e16104. doi: 10.7717/peerj.16104 (PMC10557942; doi:10.7717/peerj.16104)
Supplement: Supplemental Information 8 [file peerj-11-16104-s008.doc]

**Supplementary Table 3. The results of the KEGG enrichment analysis.**

| **ID** | **Description** | **GeneRatio** | **BgRatio** | **pvalue** | **p.adjust** | **qvalue** | **geneID** | **Count** |
| --- | --- | --- | --- | --- | --- | --- | --- | --- |
| hsa04060 | Cytokine-cytokine receptor interaction | 20/153 | 294/8075 | 0.00 | 0.000132 | 0.000113 | TNFRSF12A/CXCL8/IL13RA2/LIF/CXCL10/GDF10/GDF15/TNFRSF11B/CLCF1/CXCL9/CXCL11/IL2RA/TNFSF14/CCL20/CXCL6/CD70/CCL7/IL36B/GDF3/CSF2 | 20 |
| hsa04974 | Protein digestion and absorption | 11/153 | 103/8075 | 0.00 | 0.00038 | 0.000326 | COL4A2/COL1A2/COL4A1/COL1A1/COL3A1/COL6A2/COL5A2/COL5A1/COL6A3/COL8A1/DPP4 | 11 |
| hsa04512 | ECM-receptor interaction | 10/153 | 88/8075 | 0.00 | 0.000407 | 0.000349 | COL4A2/COL1A2/COL4A1/COL1A1/COL6A2/SV2B/THBS1/COL6A3/SDC1/IBSP | 10 |
| hsa04657 | IL-17 signaling pathway | 9/153 | 94/8075 | 0.00 | 0.003532 | 0.003029 | S100A9/CXCL8/MMP9/CXCL10/CCL20/CXCL6/MMP13/CCL7/CSF2 | 9 |
| hsa04061 | Viral protein interaction with cytokine and cytokine receptor | 9/153 | 100/8075 | 0.00 | 0.003532 | 0.003029 | CXCL8/CXCL10/CXCL9/CXCL11/IL2RA/TNFSF14/CCL20/CXCL6/CCL7 | 9 |
| hsa04933 | AGE-RAGE signaling pathway in diabetic complications | 9/153 | 100/8075 | 0.00 | 0.003532 | 0.003029 | COL4A2/COL1A2/COL4A1/COL1A1/COL3A1/VEGFA/SERPINE1/CXCL8/NOX4 | 9 |
| hsa05146 | Amoebiasis | 9/153 | 102/8075 | 0.00 | 0.003532 | 0.003029 | COL4A2/COL1A2/COL4A1/COL1A1/COL3A1/CXCL8/PRKCG/CD1A/CSF2 | 9 |
| hsa05205 | Proteoglycans in cancer | 13/153 | 205/8075 | 0.00 | 0.003532 | 0.003029 | COL1A2/CAMK2A/COL1A1/VEGFA/THBS1/PLAU/PRKCG/MMP9/LUM/SDC1/WNT10B/HOXD10/WNT16 | 13 |
| hsa04926 | Relaxin signaling pathway | 9/153 | 129/8075 | 0.00 | 0.017664 | 0.015151 | COL4A2/COL1A2/COL4A1/COL1A1/COL3A1/VEGFA/MMP9/MMP13/CREB3L3 | 9 |
| hsa05031 | Amphetamine addiction | 6/153 | 69/8075 | 0.00 | 0.040635 | 0.034852 | CAMK2A/GRIN1/PRKCG/CREB3L3/CALML3/CALML5 | 6 |
