# Supplementary material for: High LYRM4-AS1 predicts poor prognosis in patients with glioma and correlates with immune infiltration
Source: PeerJ. 2023 Oct 3;11:e16104. doi: 10.7717/peerj.16104 (PMC10557942; doi:10.7717/peerj.16104)
Supplement: Supplemental Information 10 [file peerj-11-16104-s009.doc]

**Supplementary Table 4. The results of GSEA analysis.**

| **ID** | **enrichmentScore** | **NES** | **pvalue** | **p.adjust** | **FDR** |
| --- | --- | --- | --- | --- | --- |
| REACTOME_CELL_CYCLE_MITOTIC | 0.538 | 1.613 | 0.001 | 0.02 | 0.015 |
| REACTOME_NEUTROPHIL_DEGRANULATION | 0.518 | 1.552 | 0.001 | 0.02 | 0.015 |
| REACTOME_M_PHASE | 0.506 | 1.509 | 0.001 | 0.02 | 0.015 |
| KEGG_CYTOKINE_CYTOKINE_RECEPTOR_INTERACTION | 0.613 | 1.815 | 0.001 | 0.02 | 0.015 |
| REACTOME_DNA_REPAIR | 0.49 | 1.455 | 0.001 | 0.02 | 0.015 |
| REACTOME_EXTRACELLULAR_MATRIX_ORGANIZATION | 0.636 | 1.887 | 0.001 | 0.02 | 0.015 |
| NABA_ECM_REGULATORS | 0.554 | 1.637 | 0.001 | 0.02 | 0.015 |
| NABA_CORE_MATRISOME | 0.568 | 1.685 | 0.001 | 0.02 | 0.015 |
| REACTOME_CELL_CYCLE_CHECKPOINTS | 0.591 | 1.753 | 0.001 | 0.02 | 0.015 |
| NABA_ECM_GLYCOPROTEINS | 0.539 | 1.575 | 0.001 | 0.02 | 0.015 |
| REACTOME_MITOTIC_PROMETAPHASE | 0.538 | 1.572 | 0.001 | 0.02 | 0.015 |
| REACTOME_CELL_SURFACE_INTERACTIONS_AT_THE_VASCULAR_WALL | 0.677 | 1.979 | 0.001 | 0.02 | 0.015 |
| KEGG_FOCAL_ADHESION | 0.528 | 1.544 | 0.001 | 0.02 | 0.015 |
| REACTOME_FC_EPSILON_RECEPTOR_FCERI_SIGNALING | 0.595 | 1.736 | 0.001 | 0.02 | 0.015 |
| REACTOME_INTERFERON_SIGNALING | 0.526 | 1.537 | 0.001 | 0.02 | 0.015 |
| REACTOME_IMMUNOREGULATORY_INTERACTIONS_BETWEEN_A_LYMPHOID_AND_A_NON_LYMPHOID_CELL | 0.734 | 2.14 | 0.001 | 0.02 | 0.015 |
| REACTOME_SIGNALING_BY_THE_B_CELL_RECEPTOR_BCR | 0.603 | 1.741 | 0.001 | 0.02 | 0.015 |
| REACTOME_S_PHASE | 0.556 | 1.603 | 0.001 | 0.02 | 0.015 |
| KEGG_JAK_STAT_SIGNALING_PATHWAY | 0.55 | 1.585 | 0.001 | 0.02 | 0.015 |
| REACTOME_G2_M_CHECKPOINTS | 0.613 | 1.755 | 0.001 | 0.02 | 0.015 |
| REACTOME_MITOTIC_G1_G1_S_PHASES | 0.577 | 1.654 | 0.001 | 0.02 | 0.015 |
| REACTOME_DNA_DOUBLE_STRAND_BREAK_REPAIR | 0.556 | 1.589 | 0.001 | 0.02 | 0.015 |
| REACTOME_DEGRADATION_OF_THE_EXTRACELLULAR_MATRIX | 0.632 | 1.803 | 0.001 | 0.02 | 0.015 |
| REACTOME_DNA_REPLICATION | 0.617 | 1.759 | 0.001 | 0.02 | 0.015 |
| REACTOME_FCERI_MEDIATED_NF_KB_ACTIVATION | 0.693 | 1.977 | 0.001 | 0.02 | 0.015 |
| KEGG_CELL_CYCLE | 0.624 | 1.775 | 0.001 | 0.02 | 0.015 |
| PID_P53_DOWNSTREAM_PATHWAY | 0.556 | 1.585 | 0.001 | 0.02 | 0.015 |
| REACTOME_FCGAMMA_RECEPTOR_FCGR_DEPENDENT_PHAGOCYTOSIS | 0.651 | 1.856 | 0.001 | 0.02 | 0.015 |
| REACTOME_RHO_GTPASES_ACTIVATE_FORMINS | 0.556 | 1.585 | 0.001 | 0.02 | 0.015 |
| REACTOME_COMPLEMENT_CASCADE | 0.766 | 2.166 | 0.001 | 0.02 | 0.015 |
| REACTOME_RESOLUTION_OF_SISTER_CHROMATID_COHESION | 0.594 | 1.687 | 0.001 | 0.02 | 0.015 |
| REACTOME_REGULATION_OF_ACTIN_DYNAMICS_FOR_PHAGOCYTIC_CUP_FORMATION | 0.696 | 1.97 | 0.001 | 0.02 | 0.015 |
| REACTOME_HOMOLOGY_DIRECTED_REPAIR | 0.6 | 1.698 | 0.001 | 0.02 | 0.015 |
| REACTOME_INTERLEUKIN_4_AND_INTERLEUKIN_13_SIGNALING | 0.659 | 1.861 | 0.001 | 0.02 | 0.015 |
| REACTOME_MITOTIC_SPINDLE_CHECKPOINT | 0.625 | 1.762 | 0.001 | 0.02 | 0.015 |
| KEGG_SYSTEMIC_LUPUS_ERYTHEMATOSUS | 0.678 | 1.905 | 0.001 | 0.02 | 0.015 |
| REACTOME_ANTIGEN_PROCESSING_CROSS_PRESENTATION | 0.604 | 1.69 | 0.001 | 0.02 | 0.015 |
| REACTOME_BINDING_AND_UPTAKE_OF_LIGANDS_BY_SCAVENGER_RECEPTORS | 0.791 | 2.208 | 0.001 | 0.02 | 0.015 |
| REACTOME_ACTIVATION_OF_HOX_GENES_DURING_DIFFERENTIATION | 0.648 | 1.798 | 0.001 | 0.02 | 0.015 |
| REACTOME_INTERFERON_GAMMA_SIGNALING | 0.624 | 1.731 | 0.001 | 0.02 | 0.015 |
| REACTOME_PRE_NOTCH_EXPRESSION_AND_PROCESSING | 0.587 | 1.626 | 0.001 | 0.02 | 0.015 |
| REACTOME_COLLAGEN_FORMATION | 0.714 | 1.973 | 0.001 | 0.02 | 0.015 |
| REACTOME_AMYLOID_FIBER_FORMATION | 0.617 | 1.682 | 0.001 | 0.02 | 0.015 |
| REACTOME_FCERI_MEDIATED_MAPK_ACTIVATION | 0.758 | 2.081 | 0.001 | 0.02 | 0.015 |
| REACTOME_SENESCENCE_ASSOCIATED_SECRETORY_PHENOTYPE_SASP | 0.636 | 1.736 | 0.001 | 0.02 | 0.015 |
| KEGG_ANTIGEN_PROCESSING_AND_PRESENTATION | 0.571 | 1.561 | 0.001 | 0.02 | 0.015 |
| KEGG_ECM_RECEPTOR_INTERACTION | 0.648 | 1.774 | 0.001 | 0.02 | 0.015 |
| REACTOME_ANTIGEN_ACTIVATES_B_CELL_RECEPTOR_BCR_LEADING_TO_GENERATION_OF_SECOND_MESSENGERS | 0.74 | 2.026 | 0.001 | 0.02 | 0.015 |
| REACTOME_DNA_REPLICATION_PRE_INITIATION | 0.616 | 1.688 | 0.001 | 0.02 | 0.015 |
| REACTOME_ROLE_OF_PHOSPHOLIPIDS_IN_PHAGOCYTOSIS | 0.782 | 2.127 | 0.001 | 0.02 | 0.015 |
| KEGG_HEMATOPOIETIC_CELL_LINEAGE | 0.65 | 1.783 | 0.001 | 0.02 | 0.015 |
| REACTOME_CHROMOSOME_MAINTENANCE | 0.666 | 1.827 | 0.001 | 0.02 | 0.015 |
| REACTOME_FCERI_MEDIATED_CAPLUS2_MOBILIZATION | 0.751 | 2.06 | 0.001 | 0.02 | 0.015 |
| REACTOME_INTEGRIN_CELL_SURFACE_INTERACTIONS | 0.683 | 1.873 | 0.001 | 0.02 | 0.015 |
| PID_P73PATHWAY | 0.597 | 1.619 | 0.001 | 0.02 | 0.015 |
| REACTOME_INITIAL_TRIGGERING_OF_COMPLEMENT | 0.83 | 2.249 | 0.001 | 0.02 | 0.015 |
| KEGG_COMPLEMENT_AND_COAGULATION_CASCADES | 0.623 | 1.678 | 0.001 | 0.02 | 0.015 |
| REACTOME_BASE_EXCISION_REPAIR | 0.61 | 1.642 | 0.001 | 0.02 | 0.015 |
| REACTOME_COSTIMULATION_BY_THE_CD28_FAMILY | 0.622 | 1.674 | 0.001 | 0.02 | 0.015 |
| REACTOME_CREATION_OF_C4_AND_C2_ACTIVATORS | 0.839 | 2.26 | 0.001 | 0.02 | 0.015 |
| REACTOME_ROLE_OF_LAT2_NTAL_LAB_ON_CALCIUM_MOBILIZATION | 0.812 | 2.185 | 0.001 | 0.02 | 0.015 |
| REACTOME_RUNX1_REGULATES_GENES_INVOLVED_IN_MEGAKARYOCYTE_DIFFERENTIATION_AND_PLATELET_FUNCTION | 0.602 | 1.621 | 0.001 | 0.02 | 0.015 |
| REACTOME_ECM_PROTEOGLYCANS | 0.69 | 1.866 | 0.001 | 0.02 | 0.015 |
| REACTOME_PROCESSING_OF_DNA_DOUBLE_STRAND_BREAK_ENDS | 0.618 | 1.671 | 0.001 | 0.02 | 0.015 |
| REACTOME_TRANSCRIPTIONAL_REGULATION_BY_SMALL_RNAS | 0.608 | 1.644 | 0.001 | 0.02 | 0.015 |
| KEGG_LEISHMANIA_INFECTION | 0.678 | 1.827 | 0.001 | 0.02 | 0.015 |
| REACTOME_G2_M_DNA_DAMAGE_CHECKPOINT | 0.635 | 1.715 | 0.001 | 0.02 | 0.015 |
| PID_AVB3_INTEGRIN_PATHWAY | 0.648 | 1.743 | 0.001 | 0.02 | 0.015 |
| PID_E2F_PATHWAY | 0.591 | 1.589 | 0.001 | 0.02 | 0.015 |
| PID_INTEGRIN1_PATHWAY | 0.726 | 1.938 | 0.001 | 0.02 | 0.015 |
| REACTOME_HDR_THROUGH_HOMOLOGOUS_RECOMBINATION_HRR | 0.628 | 1.676 | 0.001 | 0.02 | 0.015 |
| REACTOME_SCAVENGING_OF_HEME_FROM_PLASMA | 0.824 | 2.199 | 0.001 | 0.02 | 0.015 |
| REACTOME_FCGR_ACTIVATION | 0.823 | 2.195 | 0.001 | 0.02 | 0.015 |
| REACTOME_COLLAGEN_BIOSYNTHESIS_AND_MODIFYING_ENZYMES | 0.706 | 1.877 | 0.001 | 0.02 | 0.015 |
| REACTOME_TELOMERE_MAINTENANCE | 0.659 | 1.744 | 0.001 | 0.02 | 0.015 |
| REACTOME_ASSEMBLY_OF_COLLAGEN_FIBRILS_AND_OTHER_MULTIMERIC_STRUCTURES | 0.738 | 1.956 | 0.001 | 0.02 | 0.015 |
| REACTOME_TRANSCRIPTIONAL_REGULATION_OF_GRANULOPOIESIS | 0.668 | 1.771 | 0.001 | 0.02 | 0.015 |
| REACTOME_CD22_MEDIATED_BCR_REGULATION | 0.82 | 2.164 | 0.001 | 0.02 | 0.015 |
| REACTOME_NON_INTEGRIN_MEMBRANE_ECM_INTERACTIONS | 0.658 | 1.735 | 0.001 | 0.02 | 0.015 |
| REACTOME_COLLAGEN_DEGRADATION | 0.744 | 1.969 | 0.001 | 0.02 | 0.015 |
| REACTOME_DNA_DAMAGE_TELOMERE_STRESS_INDUCED_SENESCENCE | 0.664 | 1.742 | 0.001 | 0.02 | 0.015 |
| REACTOME_MEIOTIC_RECOMBINATION | 0.695 | 1.825 | 0.001 | 0.02 | 0.015 |
| REACTOME_NUCLEOSOME_ASSEMBLY | 0.71 | 1.83 | 0.001 | 0.02 | 0.015 |
| PID_SYNDECAN_1_PATHWAY | 0.757 | 1.944 | 0.001 | 0.02 | 0.015 |
| REACTOME_CHEMOKINE_RECEPTORS_BIND_CHEMOKINES | 0.719 | 1.846 | 0.001 | 0.02 | 0.015 |
| REACTOME_ELASTIC_FIBRE_FORMATION | 0.728 | 1.862 | 0.001 | 0.02 | 0.015 |
| NABA_COLLAGENS | 0.715 | 1.823 | 0.001 | 0.02 | 0.015 |
| PID_PLK1_PATHWAY | 0.741 | 1.89 | 0.001 | 0.02 | 0.015 |
| REACTOME_COLLAGEN_CHAIN_TRIMERIZATION | 0.715 | 1.823 | 0.001 | 0.02 | 0.015 |
| REACTOME_CONDENSATION_OF_PROPHASE_CHROMOSOMES | 0.72 | 1.836 | 0.001 | 0.02 | 0.015 |
| PID_INTEGRIN3_PATHWAY | 0.705 | 1.786 | 0.001 | 0.02 | 0.015 |
| PID_UPA_UPAR_PATHWAY | 0.708 | 1.795 | 0.001 | 0.02 | 0.015 |
| PID_FOXM1_PATHWAY | 0.766 | 1.917 | 0.001 | 0.02 | 0.015 |
| REACTOME_SIRT1_NEGATIVELY_REGULATES_RRNA_EXPRESSION | 0.675 | 1.683 | 0.001 | 0.02 | 0.015 |
| KEGG_GRAFT_VERSUS_HOST_DISEASE | 0.767 | 1.904 | 0.001 | 0.02 | 0.015 |
| PID_AURORA_B_PATHWAY | 0.775 | 1.923 | 0.001 | 0.02 | 0.015 |
| REACTOME_ACTIVATION_OF_ATR_IN_RESPONSE_TO_REPLICATION_STRESS | 0.746 | 1.852 | 0.001 | 0.02 | 0.015 |
| REACTOME_INTERLEUKIN_10_SIGNALING | 0.741 | 1.862 | 0.001 | 0.02 | 0.015 |
| REACTOME_MET_PROMOTES_CELL_MOTILITY | 0.728 | 1.83 | 0.001 | 0.02 | 0.015 |
| REACTOME_MOLECULES_ASSOCIATED_WITH_ELASTIC_FIBRES | 0.707 | 1.758 | 0.001 | 0.02 | 0.015 |
| PID_FRA_PATHWAY | 0.731 | 1.807 | 0.001 | 0.02 | 0.015 |
| REACTOME_ACTIVATION_OF_MATRIX_METALLOPROTEINASES | 0.705 | 1.715 | 0.001 | 0.02 | 0.015 |
| REACTOME_ACTIVATION_OF_THE_PRE_REPLICATIVE_COMPLEX | 0.718 | 1.751 | 0.001 | 0.02 | 0.015 |
| REACTOME_GENERATION_OF_SECOND_MESSENGER_MOLECULES | 0.734 | 1.787 | 0.001 | 0.02 | 0.015 |
| REACTOME_MET_ACTIVATES_PTK2_SIGNALING | 0.764 | 1.835 | 0.001 | 0.02 | 0.015 |
| KEGG_ALLOGRAFT_REJECTION | 0.76 | 1.868 | 0.001 | 0.02 | 0.015 |
| REACTOME_DNA_METHYLATION | 0.724 | 1.779 | 0.001 | 0.02 | 0.015 |
| KEGG_ASTHMA | 0.742 | 1.776 | 0.001 | 0.02 | 0.015 |
| REACTOME_G1_S_SPECIFIC_TRANSCRIPTION | 0.722 | 1.734 | 0.001 | 0.02 | 0.015 |
| REACTOME_SYNDECAN_INTERACTIONS | 0.747 | 1.784 | 0.001 | 0.02 | 0.015 |
| REACTOME_CYCLIN_A_B1_B2_ASSOCIATED_EVENTS_DURING_G2_M_TRANSITION | 0.789 | 1.855 | 0.001 | 0.02 | 0.015 |
| BIOCARTA_NKT_PATHWAY | 0.727 | 1.7 | 0.001 | 0.02 | 0.015 |
| PID_INTEGRIN_A9B1_PATHWAY | 0.756 | 1.767 | 0.001 | 0.02 | 0.015 |
| BIOCARTA_INFLAM_PATHWAY | 0.737 | 1.735 | 0.001 | 0.02 | 0.015 |
| REACTOME_PD_1_SIGNALING | 0.816 | 1.895 | 0.001 | 0.02 | 0.015 |
| BIOCARTA_CASPASE_PATHWAY | 0.734 | 1.693 | 0.001 | 0.02 | 0.015 |
| REACTOME_PHOSPHORYLATION_OF_CD3_AND_TCR_ZETA_CHAINS | 0.845 | 1.949 | 0.001 | 0.02 | 0.015 |
| BIOCARTA_CTLA4_PATHWAY | 0.78 | 1.763 | 0.001 | 0.02 | 0.015 |
| REACTOME_SCAVENGING_BY_CLASS_A_RECEPTORS | 0.761 | 1.694 | 0.001 | 0.02 | 0.015 |
| REACTOME_TRANSLOCATION_OF_ZAP_70_TO_IMMUNOLOGICAL_SYNAPSE | 0.849 | 1.89 | 0.001 | 0.02 | 0.015 |
| REACTOME_CROSSLINKING_OF_COLLAGEN_FIBRILS | 0.768 | 1.703 | 0.001 | 0.02 | 0.015 |
| BIOCARTA_CTL_PATHWAY | 0.863 | 1.798 | 0.001 | 0.02 | 0.015 |
| BIOCARTA_TCRA_PATHWAY | 0.845 | 1.733 | 0.001 | 0.02 | 0.015 |
| BIOCARTA_TCYTOTOXIC_PATHWAY | 0.847 | 1.738 | 0.001 | 0.02 | 0.015 |
| REACTOME_CONDENSATION_OF_PROMETAPHASE_CHROMOSOMES | 0.847 | 1.694 | 0.001 | 0.021 | 0.016 |
| REACTOME_RHO_GTPASE_EFFECTORS | 0.468 | 1.389 | 0.002 | 0.029 | 0.022 |
| REACTOME_MITOTIC_METAPHASE_AND_ANAPHASE | 0.515 | 1.505 | 0.002 | 0.029 | 0.022 |
| REACTOME_CELLULAR_SENESCENCE | 0.529 | 1.528 | 0.002 | 0.029 | 0.022 |
| REACTOME_MITOTIC_PROPHASE | 0.562 | 1.59 | 0.002 | 0.029 | 0.022 |
| REACTOME_RUNX1_REGULATES_TRANSCRIPTION_OF_GENES_INVOLVED_IN_DIFFERENTIATION_OF_HSCS | 0.579 | 1.63 | 0.002 | 0.029 | 0.022 |
| REACTOME_ORC1_REMOVAL_FROM_CHROMATIN | 0.591 | 1.59 | 0.002 | 0.03 | 0.023 |
| REACTOME_ACTIVATION_OF_APC_C_AND_APC_C:CDC20_MEDIATED_DEGRADATION_OF_MITOTIC_PROTEINS | 0.576 | 1.554 | 0.002 | 0.03 | 0.023 |
| PID_IL12_2PATHWAY | 0.635 | 1.684 | 0.002 | 0.03 | 0.023 |
| REACTOME_SIGNALING_BY_PDGF | 0.631 | 1.663 | 0.002 | 0.03 | 0.023 |
| KEGG_AUTOIMMUNE_THYROID_DISEASE | 0.653 | 1.683 | 0.002 | 0.031 | 0.023 |
| KEGG_INTESTINAL_IMMUNE_NETWORK_FOR_IGA_PRODUCTION | 0.671 | 1.718 | 0.002 | 0.031 | 0.023 |
| REACTOME_PRC2_METHYLATES_HISTONES_AND_DNA | 0.67 | 1.71 | 0.002 | 0.031 | 0.023 |
| BIOCARTA_IL2RB_PATHWAY | 0.672 | 1.668 | 0.002 | 0.031 | 0.023 |
| KEGG_DNA_REPLICATION | 0.677 | 1.673 | 0.002 | 0.031 | 0.023 |
| REACTOME_DEPURINATION | 0.676 | 1.656 | 0.002 | 0.031 | 0.023 |
| REACTOME_DNA_STRAND_ELONGATION | 0.693 | 1.687 | 0.002 | 0.031 | 0.023 |
| REACTOME_RESOLUTION_OF_D_LOOP_STRUCTURES | 0.685 | 1.67 | 0.002 | 0.031 | 0.023 |
| REACTOME_LAMININ_INTERACTIONS | 0.702 | 1.686 | 0.002 | 0.031 | 0.023 |
| KEGG_PRIMARY_IMMUNODEFICIENCY | 0.667 | 1.639 | 0.002 | 0.031 | 0.023 |
| REACTOME_G0_AND_EARLY_G1 | 0.718 | 1.713 | 0.002 | 0.031 | 0.023 |
| REACTOME_RESOLUTION_OF_D_LOOP_STRUCTURES_THROUGH_SYNTHESIS_DEPENDENT_STRAND_ANNEALING_SDSA | 0.709 | 1.667 | 0.003 | 0.031 | 0.023 |
| BIOCARTA_TH1TH2_PATHWAY | 0.722 | 1.65 | 0.003 | 0.032 | 0.024 |
| REACTOME_POLO_LIKE_KINASE_MEDIATED_EVENTS | 0.811 | 1.76 | 0.003 | 0.033 | 0.025 |
| BIOCARTA_BLYMPHOCYTE_PATHWAY | 0.832 | 1.707 | 0.003 | 0.034 | 0.025 |
| BIOCARTA_MHC_PATHWAY | 0.84 | 1.722 | 0.003 | 0.034 | 0.025 |
| BIOCARTA_THELPER_PATHWAY | 0.839 | 1.72 | 0.003 | 0.034 | 0.025 |
| NABA_SECRETED_FACTORS | 0.455 | 1.354 | 0.003 | 0.035 | 0.027 |
| BIOCARTA_GABA_PATHWAY | -0.88 | -2.021 | 0.003 | 0.035 | 0.027 |
| REACTOME_NEUROTOXICITY_OF_CLOSTRIDIUM_TOXINS | -0.895 | -2.055 | 0.003 | 0.035 | 0.027 |
| BIOCARTA_BARRESTIN_PATHWAY | -0.824 | -1.976 | 0.003 | 0.036 | 0.027 |
| KEGG_CELL_ADHESION_MOLECULES_CAMS | 0.532 | 1.516 | 0.003 | 0.036 | 0.027 |
| REACTOME_REPRODUCTION | 0.529 | 1.494 | 0.003 | 0.036 | 0.027 |
| REACTOME_MEIOSIS | 0.571 | 1.582 | 0.003 | 0.036 | 0.027 |
| REACTOME_SWITCHING_OF_ORIGINS_TO_A_POST_REPLICATIVE_STATE | 0.577 | 1.599 | 0.003 | 0.036 | 0.027 |
| REACTOME_NEGATIVE_EPIGENETIC_REGULATION_OF_RRNA_EXPRESSION | 0.553 | 1.51 | 0.003 | 0.036 | 0.027 |
| KEGG_SMALL_CELL_LUNG_CANCER | 0.573 | 1.571 | 0.003 | 0.036 | 0.027 |
| REACTOME_REGULATION_OF_MITOTIC_CELL_CYCLE | 0.575 | 1.576 | 0.003 | 0.036 | 0.027 |
| KEGG_P53_SIGNALING_PATHWAY | 0.605 | 1.612 | 0.003 | 0.036 | 0.027 |
| PID_IL4_2PATHWAY | 0.63 | 1.674 | 0.003 | 0.036 | 0.027 |
| REACTOME_FORMATION_OF_THE_BETA_CATENIN:TCF_TRANSACTIVATING_COMPLEX | 0.628 | 1.663 | 0.003 | 0.036 | 0.027 |
| REACTOME_HDACS_DEACETYLATE_HISTONES | 0.606 | 1.605 | 0.003 | 0.036 | 0.027 |
| REACTOME_PRESYNAPTIC_DEPOLARIZATION_AND_CALCIUM_CHANNEL_OPENING | -0.876 | -2.113 | 0.003 | 0.036 | 0.027 |
| REACTOME_SEROTONIN_RECEPTORS | -0.802 | -1.934 | 0.003 | 0.036 | 0.027 |
| REACTOME_RMTS_METHYLATE_HISTONE_ARGININES | 0.61 | 1.586 | 0.003 | 0.036 | 0.027 |
| REACTOME_ERCC6_CSB_AND_EHMT2_G9A_POSITIVELY_REGULATE_RRNA_EXPRESSION | 0.645 | 1.657 | 0.004 | 0.036 | 0.027 |
| NABA_BASEMENT_MEMBRANES | 0.655 | 1.641 | 0.004 | 0.036 | 0.027 |
| PID_ATR_PATHWAY | 0.661 | 1.647 | 0.004 | 0.036 | 0.027 |
| REACTOME_DAP12_INTERACTIONS | 0.651 | 1.621 | 0.004 | 0.036 | 0.027 |
| BIOCARTA_BARR_MAPK_PATHWAY | -0.796 | -1.93 | 0.004 | 0.036 | 0.027 |
| REACTOME_ACTIVATED_PKN1_STIMULATES_TRANSCRIPTION_OF_AR_ANDROGEN_RECEPTOR_REGULATED_GENES_KLK2_AND_KLK3 | 0.66 | 1.64 | 0.004 | 0.036 | 0.027 |
| BIOCARTA_CK1_PATHWAY | -0.761 | -1.927 | 0.004 | 0.036 | 0.027 |
| BIOCARTA_PGC1A_PATHWAY | -0.817 | -2.071 | 0.004 | 0.036 | 0.027 |
| PID_LPA4_PATHWAY | -0.751 | -1.902 | 0.004 | 0.036 | 0.027 |
| REACTOME_LGI_ADAM_INTERACTIONS | -0.854 | -2.112 | 0.004 | 0.036 | 0.027 |
| PID_SYNDECAN_4_PATHWAY | 0.675 | 1.643 | 0.004 | 0.036 | 0.027 |
| PID_IL2_STAT5_PATHWAY | 0.672 | 1.614 | 0.004 | 0.036 | 0.027 |
| REACTOME_ACETYLCHOLINE_NEUROTRANSMITTER_RELEASE_CYCLE | -0.783 | -2.023 | 0.004 | 0.036 | 0.027 |
| REACTOME_CGMP_EFFECTS | -0.73 | -1.885 | 0.004 | 0.036 | 0.027 |
| REACTOME_EXTENSION_OF_TELOMERES | 0.681 | 1.636 | 0.004 | 0.036 | 0.027 |
| REACTOME_INTERLEUKIN_7_SIGNALING | 0.698 | 1.671 | 0.004 | 0.036 | 0.027 |
| BIOCARTA_PDZS_PATHWAY | -0.786 | -2.059 | 0.004 | 0.036 | 0.027 |
| REACTOME_NOREPINEPHRINE_NEUROTRANSMITTER_RELEASE_CYCLE | -0.861 | -2.255 | 0.004 | 0.036 | 0.027 |
| REACTOME_SEROTONIN_NEUROTRANSMITTER_RELEASE_CYCLE | -0.908 | -2.379 | 0.004 | 0.036 | 0.027 |
| REACTOME_TRAFFICKING_OF_GLUR2_CONTAINING_AMPA_RECEPTORS | -0.753 | -1.973 | 0.004 | 0.036 | 0.027 |
| REACTOME_GABA_SYNTHESIS_RELEASE_REUPTAKE_AND_DEGRADATION | -0.862 | -2.322 | 0.004 | 0.036 | 0.027 |
| REACTOME_PKA_MEDIATED_PHOSPHORYLATION_OF_CREB | -0.703 | -1.894 | 0.004 | 0.036 | 0.027 |
| REACTOME_RAS_ACTIVATION_UPON_CA2PLUS_INFLUX_THROUGH_NMDA_RECEPTOR | -0.81 | -2.181 | 0.004 | 0.036 | 0.027 |
| REACTOME_SIGNALING_BY_NOTCH | 0.465 | 1.365 | 0.004 | 0.037 | 0.028 |
| REACTOME_NEGATIVE_REGULATION_OF_NMDA_RECEPTOR_MEDIATED_NEURONAL_TRANSMISSION | -0.839 | -2.288 | 0.004 | 0.037 | 0.028 |
| REACTOME_UNBLOCKING_OF_NMDA_RECEPTORS_GLUTAMATE_BINDING_AND_ACTIVATION | -0.836 | -2.28 | 0.004 | 0.037 | 0.028 |
| REACTOME_ESR_MEDIATED_SIGNALING | 0.464 | 1.356 | 0.004 | 0.037 | 0.028 |
| BIOCARTA_IL17_PATHWAY | 0.813 | 1.719 | 0.004 | 0.037 | 0.028 |
| REACTOME_TCR_SIGNALING | 0.509 | 1.439 | 0.004 | 0.038 | 0.029 |
| REACTOME_UNWINDING_OF_DNA | 0.8 | 1.64 | 0.004 | 0.038 | 0.029 |
| REACTOME_TRANSCRIPTIONAL_REGULATION_BY_RUNX2 | 0.503 | 1.425 | 0.004 | 0.038 | 0.029 |
| BIOCARTA_NOS1_PATHWAY | -0.801 | -2.193 | 0.004 | 0.038 | 0.029 |
| REACTOME_SYNAPTIC_ADHESION_LIKE_MOLECULES | -0.76 | -2.081 | 0.004 | 0.038 | 0.029 |
| REACTOME_IRAK4_DEFICIENCY_TLR2_4 | 0.808 | 1.616 | 0.004 | 0.039 | 0.029 |
| REACTOME_DOPAMINE_NEUROTRANSMITTER_RELEASE_CYCLE | -0.874 | -2.403 | 0.004 | 0.039 | 0.03 |
| REACTOME_LONG_TERM_POTENTIATION | -0.819 | -2.251 | 0.004 | 0.039 | 0.03 |
| REACTOME_GLUTAMATE_NEUROTRANSMITTER_RELEASE_CYCLE | -0.898 | -2.492 | 0.005 | 0.04 | 0.03 |
| KEGG_TYPE_I_DIABETES_MELLITUS | 0.646 | 1.623 | 0.005 | 0.042 | 0.031 |
| REACTOME_BASE_EXCISION_REPAIR_AP_SITE_FORMATION | 0.639 | 1.605 | 0.005 | 0.042 | 0.031 |
| REACTOME_HOMOLOGOUS_DNA_PAIRING_AND_STRAND_EXCHANGE | 0.649 | 1.63 | 0.005 | 0.042 | 0.031 |
| BIOCARTA_G1_PATHWAY | 0.688 | 1.646 | 0.005 | 0.042 | 0.032 |
| PID_INTEGRIN2_PATHWAY | 0.694 | 1.657 | 0.005 | 0.042 | 0.032 |
| REACTOME_METALLOPROTEASE_DUBS | 0.677 | 1.616 | 0.005 | 0.042 | 0.032 |
| REACTOME_CREB1_PHOSPHORYLATION_THROUGH_NMDA_RECEPTOR_MEDIATED_ACTIVATION_OF_RAS_SIGNALING | -0.709 | -2.014 | 0.005 | 0.042 | 0.032 |
| PID_LYMPH_ANGIOGENESIS_PATHWAY | 0.69 | 1.614 | 0.005 | 0.042 | 0.032 |
| REACTOME_CA_DEPENDENT_EVENTS | -0.689 | -2.154 | 0.005 | 0.042 | 0.032 |
| REACTOME_APC_C:CDC20_MEDIATED_DEGRADATION_OF_CYCLIN_B | 0.704 | 1.634 | 0.005 | 0.042 | 0.032 |
| REACTOME_GLUTAMATE_BINDING_ACTIVATION_OF_AMPA_RECEPTORS_AND_SYNAPTIC_PLASTICITY | -0.758 | -2.272 | 0.005 | 0.042 | 0.032 |
| REACTOME_E2F_MEDIATED_REGULATION_OF_DNA_REPLICATION | 0.708 | 1.633 | 0.005 | 0.042 | 0.032 |
| REACTOME_OXIDATIVE_STRESS_INDUCED_SENESCENCE | 0.539 | 1.509 | 0.005 | 0.044 | 0.033 |
| BIOCARTA_DC_PATHWAY | 0.753 | 1.659 | 0.005 | 0.044 | 0.033 |
| REACTOME_SIGNALING_BY_MET | 0.546 | 1.49 | 0.005 | 0.045 | 0.034 |
| BIOCARTA_ASBCELL_PATHWAY | 0.802 | 1.671 | 0.006 | 0.045 | 0.034 |
| REACTOME_ASSEMBLY_OF_THE_PRE_REPLICATIVE_COMPLEX | 0.585 | 1.561 | 0.006 | 0.045 | 0.034 |
| REACTOME_TRYPTOPHAN_CATABOLISM | 0.78 | 1.6 | 0.006 | 0.045 | 0.034 |
| REACTOME_AMINE_LIGAND_BINDING_RECEPTORS | -0.703 | -2.266 | 0.006 | 0.045 | 0.034 |
| REACTOME_ASSEMBLY_AND_CELL_SURFACE_PRESENTATION_OF_NMDA_RECEPTORS | -0.573 | -1.849 | 0.006 | 0.045 | 0.034 |
| REACTOME_DAG_AND_IP3_SIGNALING | -0.723 | -2.304 | 0.006 | 0.046 | 0.034 |
| REACTOME_TP53_REGULATES_TRANSCRIPTION_OF_CELL_CYCLE_GENES | 0.611 | 1.568 | 0.006 | 0.046 | 0.035 |
| REACTOME_VOLTAGE_GATED_POTASSIUM_CHANNELS | -0.803 | -2.598 | 0.006 | 0.047 | 0.036 |
| REACTOME_ADENYLATE_CYCLASE_ACTIVATING_PATHWAY | -0.82 | -1.884 | 0.006 | 0.047 | 0.036 |
| REACTOME_DISEASES_ASSOCIATED_WITH_GLYCOSAMINOGLYCAN_METABOLISM | 0.623 | 1.566 | 0.006 | 0.047 | 0.036 |
| REACTOME_TRANSCRIPTIONAL_REGULATION_BY_RUNX1 | 0.461 | 1.352 | 0.006 | 0.047 | 0.036 |
| REACTOME_TNFS_BIND_THEIR_PHYSIOLOGICAL_RECEPTORS | 0.662 | 1.58 | 0.006 | 0.048 | 0.036 |
| REACTOME_TELOMERE_C_STRAND_LAGGING_STRAND_SYNTHESIS | 0.689 | 1.611 | 0.006 | 0.048 | 0.036 |
| REACTOME_PHASE_0_RAPID_DEPOLARISATION | -0.684 | -2.242 | 0.006 | 0.048 | 0.036 |
| PID_INTEGRIN_CS_PATHWAY | 0.671 | 1.579 | 0.006 | 0.048 | 0.036 |
