# Supplementary material for: High LYRM4-AS1 predicts poor prognosis in patients with glioma and correlates with immune infiltration
Source: PeerJ. 2023 Oct 3;11:e16104. doi: 10.7717/peerj.16104 (PMC10557942; doi:10.7717/peerj.16104)
Supplement: Supplemental Information 11 [file peerj-11-16104-s010.doc]

**Supplementary Table 5. Correlation of LYRM4-AS1 expression with** **immune checkpoints.**

| **Immune checkpoint** | **R value** | **P value** |
| --- | --- | --- |
| PDCD1 | 0.44718946 | 1.2792E-35 |
| CD274 | 0.43215606 | 0 |
| CTLA4 | 0.32684184 | 7.7074E-19 |
| LAG3 | 0.27403763 | 2.1708E-13 |
| TIGIT | 0.21162733 | 1.7886E-08 |
| HAVCR2 | 0.44640389 | 0 |
