# Supplementary material for: High LYRM4-AS1 predicts poor prognosis in patients with glioma and correlates with immune infiltration
Source: PeerJ. 2023 Oct 3;11:e16104. doi: 10.7717/peerj.16104 (PMC10557942; doi:10.7717/peerj.16104)
Supplement: Supplemental Information 16 [file peerj-11-16104-s015.pdf]

0H
U87 MG

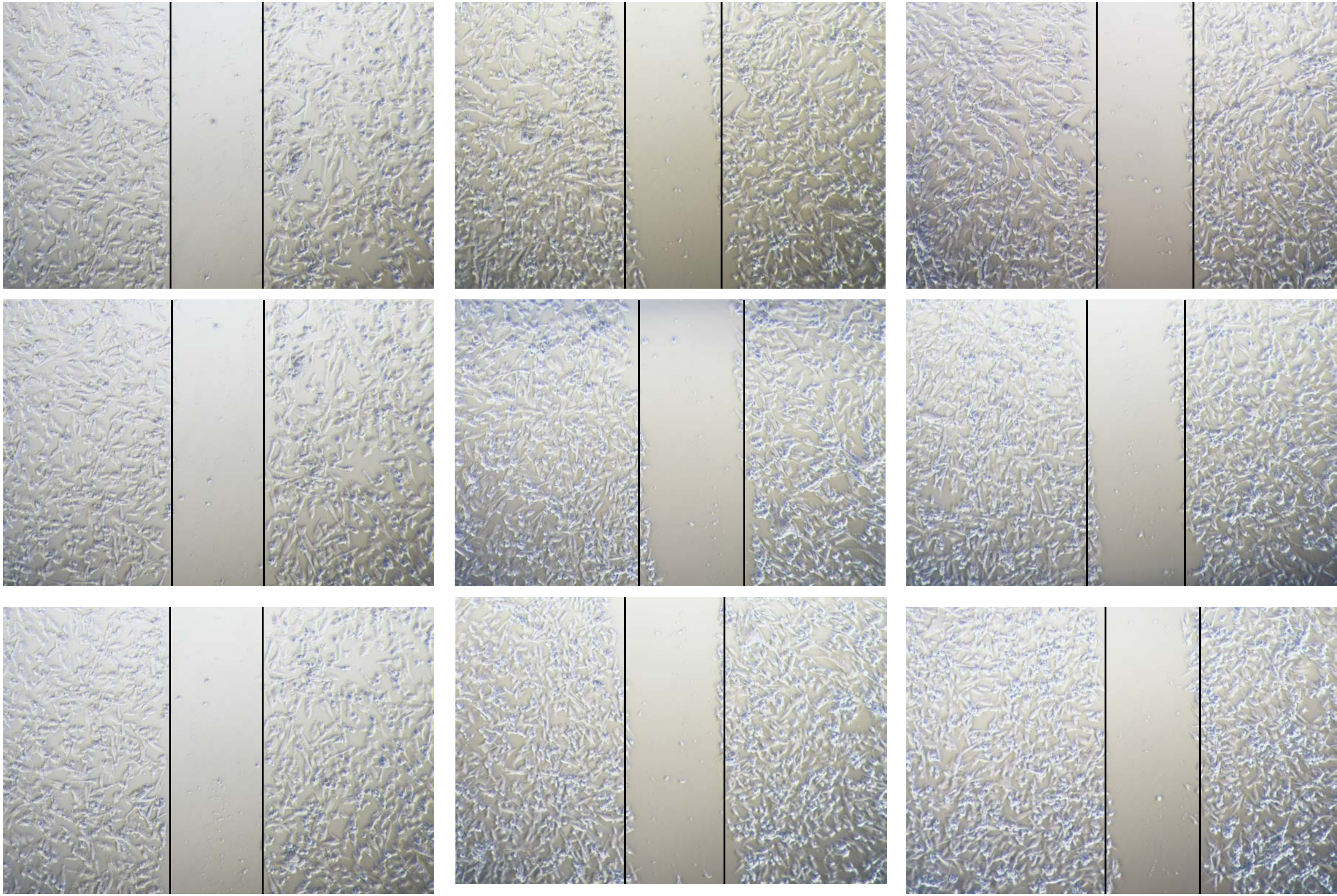

siNC

siLYR
M4-
AS1-1

siLYR
M4-
AS1-2

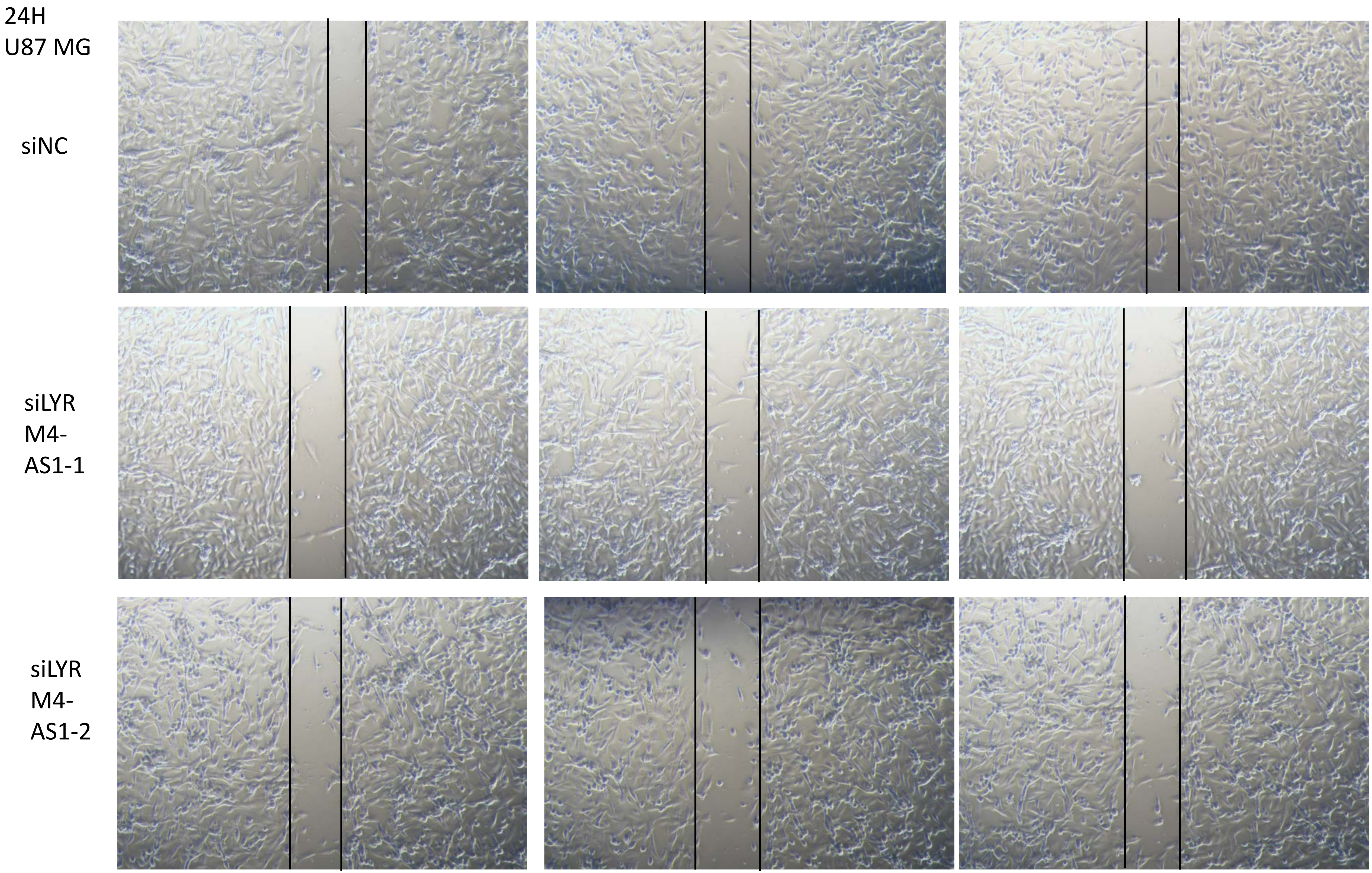
24H
U87 MG
siNC
siLYR
M4-
AS1-1
siLYR
M4-
AS1-2

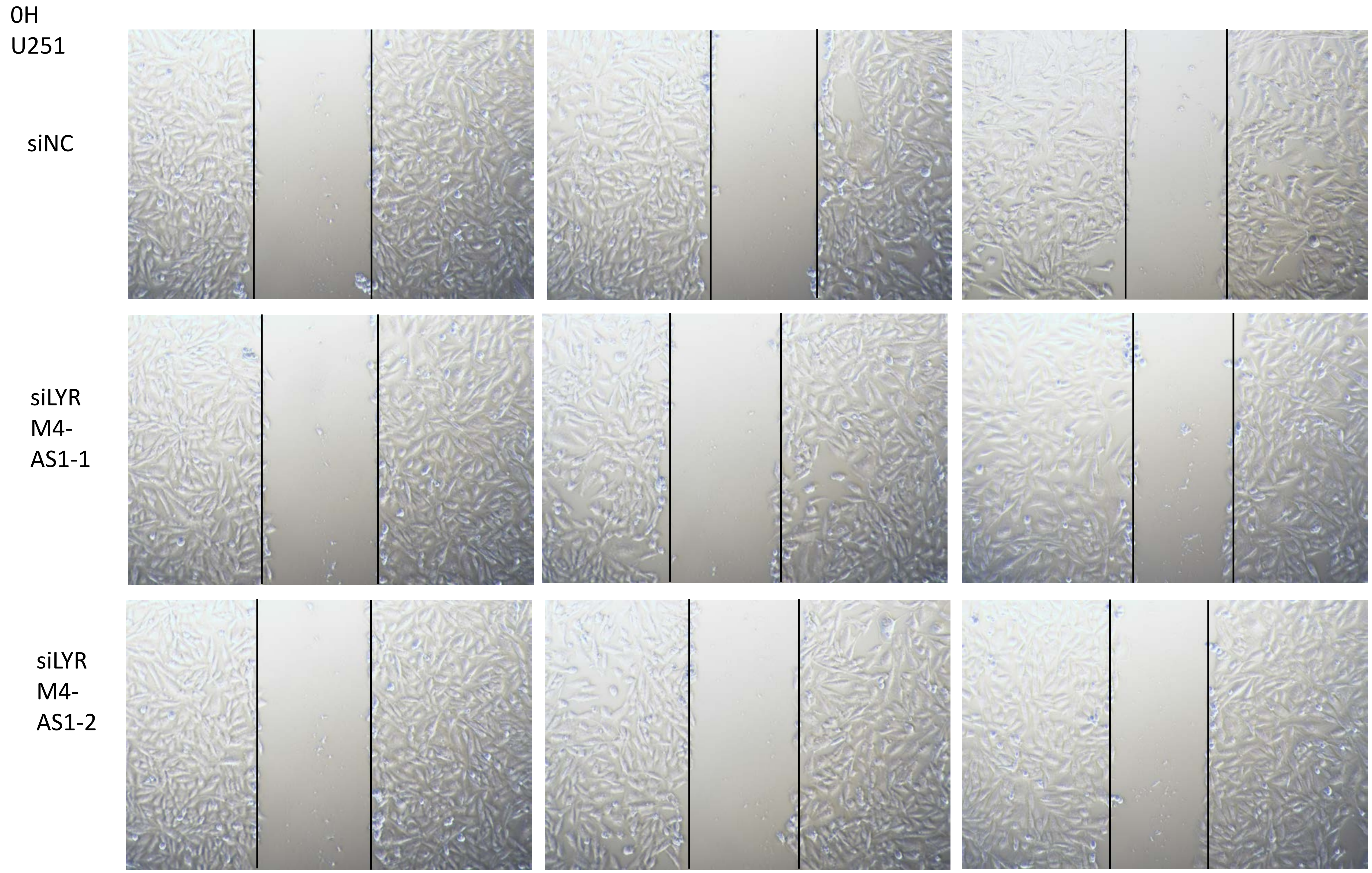
0H
U251
siNC
siLYR
M4-
AS1-1
siLYR
M4-
AS1-2

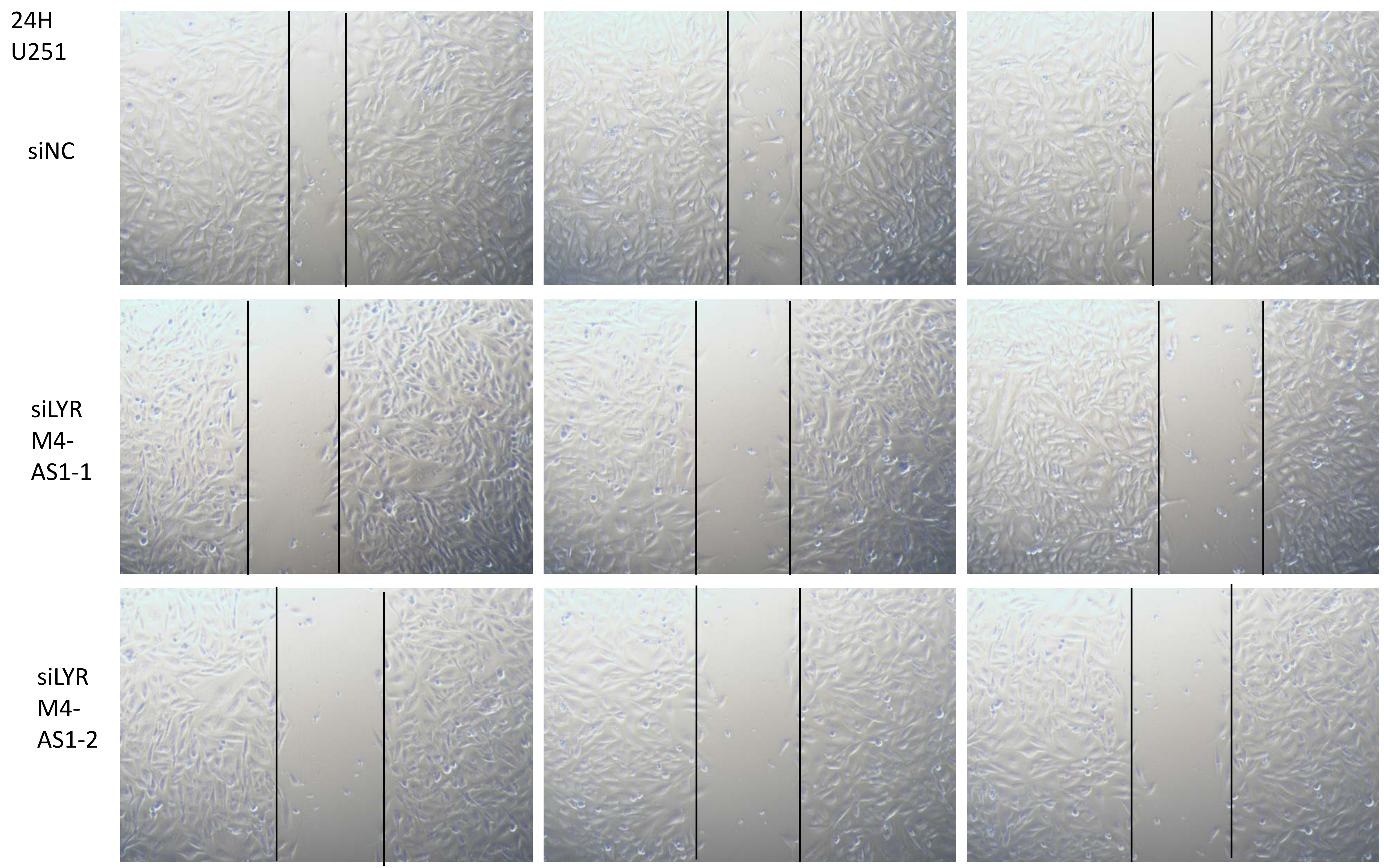
24H
U251
siNC
siLYR
M4-
AS1-1
siLYR
M4-
AS1-2
